# Electroless Nickel Plating of Material Surfaces for Ultracold Neutron Storage Traps

**DOI:** 10.3390/nano16191240

**Published:** 2026-09-30

**Authors:** K. Turlybekuly, E. Fesik, A. Muzychka, V. V. Nesvizhevsky, E. Lychagin, K. Zhernenkov, Zh. Kurmanaliyev, A. Nezvanov, T. Vershinina, R. Kiryanov, A. Koretskiy, E. Korobkina

**Affiliations:** 1 Institute of Nuclear Physics, Ibragimova Str. 1, Almaty 0500032, Kazakhstan; 2 Frank Laboratory of Neutron Physics, Joint Institute for Nuclear Research, 6 Joliot Curie, Dubna 141980, Russia; 3 Faculty of Technology of Inorganic Substances and High-Temperature Materials, D. Mendeleev University of Chemical Technology, Moscow 125047, Russia; 4 Institut Max von Laue-Paul Langevin, 71 Av. des Martyrs, F-38042 Grenoble, France; 5 Department of Nuclear Engineering, North Carolina State University, Raleigh, NC 27695, USA

**Keywords:** zirconium, aluminum, beryllium, electroless nickel plating, nickel-phosphorus coating, neutrons, ultracold neutrons, very cold neutrons

## Abstract

Electroless nickel plating of various alloys is one of the most technologically mature methods for producing functional coatings with high corrosion resistance, wear resistance, and stable service performance. In the field of ultracold neutron (UCN) physics, nickel-phosphorus coatings based on the ^58^Ni isotope are of particular interest. Its exceptionally high neutron optical potential, approximately 342 neV, is substantially higher than that of natural nickel, which is approximately 245.5 neV. At the same time, ^58^Ni and natural nickel have essentially identical chemical properties and differ only in their nuclear characteristics; therefore, process development and optimization of the deposition procedure can be performed using the more readily available natural nickel. The incorporation of phosphorus decreases the total neutron optical potential to values in the range of 190–220 neV, but makes it possible to modify the magnetic properties of the nickel coating in order to reduce magnetic losses of UCN upon reflection from the surface. Although extensive studies have been devoted to the deposition of Ni-P coatings on aluminum and steel substrates, the formation of high-quality coatings on beryllium- and zirconium-based alloys remains insufficiently investigated. These materials are of particular interest for chambers of superfluid-helium-based UCN sources, since they can reduce activation-related heat loads and ensure efficient cooling of the source to the required temperatures of approximately 1 K under high-radiation-flux conditions. In this work, electroless Ni–P coatings deposited on the surfaces of the aluminum–magnesium alloy AMg6, the aluminum–beryllium alloy AM162H, and the zirconium alloy Zircaloy-4 were systematically investigated, with particular emphasis on AM162H and Zircaloy-4 as promising structural materials for UCN-source chambers. The deposited coatings were comprehensively characterized by X-ray diffraction (XRD), scanning electron microscopy (SEM), energy-dispersive X-ray spectroscopy (EDS), inductively coupled plasma optical emission spectrometry (ICP-OES), surface profilometry, instrumented nanoindentation, scratch testing, and electrochemical polarization measurements followed by Tafel analysis. These complementary techniques provided information on the phase-structural state, surface morphology, continuity, thickness, elemental composition, roughness, hardness, elastic modulus, indentation stiffness, adhesion behavior, and corrosion resistance of the coatings. The ICP-OES measurements independently confirmed the compositional trend established by EDS. The coatings deposited on AM162H and Zircaloy-4 exhibited higher hardness and substantially better corrosion resistance than Ni–P/AMg6. Scratch testing indicated satisfactory interfacial adhesion for the coatings on AMg6 and AM162H, whereas the Ni–P/Zircaloy-4 coating exhibited comparatively weaker adhesion to the substrate under the applied test conditions. Particular attention was paid to coating continuity, composition, and thickness because through-thickness defects would expose the substrate, which has a lower neutron optical potential, impurity elements could increase UCN absorption and reduce the neutron optical potential of the coating, and an appropriately selected coating thickness would enable the rational use of the expensive ^58^Ni isotope. The results provide an experimental basis for further optimization of electroless Ni–P coatings for components of UCN production and storage systems.

## 1. Introduction

In recent decades, ultracold neutron (UCN) sources have been actively developed and constructed at leading scientific centers worldwide [1,2,3,4,5,6,7,8,9]. These sources are intended for searches for new fundamental interactions and phenomena in nature, including possible manifestations of dark matter and dark energy, as well as for high-precision studies of the fundamental properties of the neutron [10]. UCNs have extremely low kinetic energies, on the order of several tens of nanoelectronvolts, which enables them to undergo multiple reflections from material surfaces and to be stored for long periods in material, magnetic, and gravitational traps [11,12,13]. Owing to these properties, UCNs can be used, in particular, for high-precision measurements of quantum states of neutrons moving in the whispering-gallery regime [14], as well as for studies of gravitational quantum states of the neutron [15]. These experimental approaches are planned to be implemented at new very cold neutron (VCN) and UCN sources [16] being developed at the WWR-K (Water-Water Reactor–Kazakhstan) research reactor of the Institute of Nuclear Physics (INP) in Almaty. They are aimed at searches for additional fundamental interactions, both spin-dependent and spin-independent, possible manifestations of dark matter and dark energy, searches for neutron–antineutron oscillations and a nonzero electric charge of the neutron, measurements of the gravitational constant, and other experiments [17,18,19,20,21,22,23,24,25,26,27].

The efficiency of such studies directly depends on the total number of UCNs available in experimental setups. Therefore, in the development and construction of new UCN sources, particular attention is paid not only to the method of producing low-energy neutrons and to the choice of moderator, but also to all components used for UCN accumulation and extraction. Neutron losses in the accumulation chamber and in the walls of UCN guides can substantially reduce the total number of UCNs. In this respect, the material of the surface with which the neutron interacts becomes one of the key elements of the entire facility.

One of the promising projects is the development of a UCN source at the WWR-K research reactor of the INP in Almaty [16,28]. The considered source concept is based on the use of superfluid helium as a medium for UCN production [29]. In such a configuration, the superfluid-helium chamber must be located close to the reactor core, i.e., in a region with a high neutron flux and, consequently, significant thermal and radiation loads [30]. This circumstance substantially complicates the choice of structural materials and coatings, since the materials of the accumulation chamber walls and UCN guides must satisfy a set of stringent requirements. They must retain leak tightness at low temperatures, have sufficient mechanical strength, thermal conductivity, and radiation resistance, and also possess favorable neutron-optical properties. For UCNs, a high neutron-nuclei optical potential of the surface, a low probability of UCN wall losses, low neutron absorption, and weak scattering from surface inhomogeneities, roughness, and contamination are especially important [31]. Thus, the choice of wall material and coating cannot be regarded as a conventional technological task. It forms part of the broader physics and engineering problem of developing an efficient UCN source.

Aluminum alloys, zirconium alloys, including Zircaloy-4, and the AM162H aluminum–beryllium alloy, commercially known as AlBeMet, are considered as possible structural materials for components of the UCN source at the WWR-K reactor. This choice is determined not only by their structural and technological characteristics, but also by the neutron-physical requirements imposed on materials located near the superfluid-helium UCN converter. For superfluid-helium-based UCN sources, it is essential to minimize the heat load on the source chamber, since a low level of heat generation facilitates cooling of the system and makes it possible to maintain the superfluid-helium temperature about 1 K, which is optimal for efficient UCN accumulation and storage. A significant contribution to the heat load may arise from the chamber-wall material, in which radiative neutron capture and induced radioactivity occur under neutron irradiation. These processes are accompanied by energy release, including heat generation associated with the β-decay of activation products. In this regard, beryllium- and zirconium-based materials are of particular interest, since they are characterized by a more favorable combination of low neutron absorption, radiation resistance, and reduced contribution to heat generation associated with induced radioactivity compared with a number of conventional structural materials. Each of these materials has its own advantages. Aluminum alloys are attractive in terms of machinability, low density, and applicability in cryogenic assemblies. Zirconium alloys are well known as materials capable of operating in intense neutron fields [32]. Aluminum–beryllium alloys are of interest owing to their combination of low density and high stiffness [33]. However, these materials by themselves do not provide optimal conditions for UCN reflection and storage. Therefore, it is necessary to form a special surface layer with a higher neutron-nuclei optical potential and low UCN wall losses.

One of the most promising functional layers for UCN source components is a nickel-phosphorus coating, which, in the final application, can be produced using the ^58^Ni isotope. Interest in this material is associated with the high neutron-nuclei optical potential of ^58^Ni [34], reaching approximately 342 neV [35], which makes it especially attractive for efficient UCN reflection. At the same time, the use of pure nickel is limited by its ferromagnetic properties, which is particularly important for experiments with polarized UCNs. The incorporation of phosphorus into the nickel matrix makes it possible not only to obtain dense, chemically resistant, and technologically reproducible coatings by electroless deposition, but also to deliberately modify the phase-structural and magnetic state of the layer. As the phosphorus content increases, the long-range magnetic order of nickel is disrupted, the coating transforms into an amorphous or weakly crystallized state, and it may lose its ferromagnetic properties. This is of fundamental importance for neutron-optical components operating with polarized neutrons. However, an increase in the phosphorus fraction also changes the neutron-optical characteristics of the coating, including a decrease in the effective neutron-nuclei optical potential of the layer. Therefore, the composition of a Ni-P coating should be regarded as a compromise between nonmagnetic behavior, corrosion resistance, mechanical stability, and the ability to efficiently reflect UCNs. The practical applicability of such coatings for UCN physics was demonstrated by Pattie et al. [36]: for electrolessly deposited Ni-P coatings on aluminum and steel substrates with a phosphorus mass fraction of approximately 10.5%, a neutron-nuclei optical potential of about 213 neV was determined, and low effective UCN wall losses were demonstrated. Later, Akatsuka et al. [37], who investigated Ni-P coatings from different manufacturers deposited on polished aluminum and stainless-steel substrates, showed that at a phosphorus content above 10 wt.%, the coating becomes nonmagnetic. This confirmed the possibility of using such coatings in UCN guides, accumulation chambers of UCN facilities, and experiments with polarized neutrons. Since natural nickel and the ^58^Ni isotope have identical chemical properties and differ mainly in their nuclear characteristics, it is reasonable to develop electroless deposition processes, form strongly adherent layers, and evaluate the quality of Ni-P coatings using the more readily available natural nickel, whereas the transition to isotopically enriched ^58^Ni can be considered at later stages aimed at increasing the neutron-nuclei optical potential of the coating.

Recent studies demonstrate growing interest in the surface modification of Zircaloy-4 and related zirconium alloys for nuclear applications. Advanced surface-engineering approaches, including microarc-oxidized ceramic layers, electrodeposited chromium coatings, and multilayer metallic systems, have been investigated to improve corrosion resistance, mechanical durability, and interfacial stability [38,39,40,41]. These studies show that the functional performance of coated zirconium substrates is strongly affected by surface pretreatment, the condition of the native oxide layer, coating continuity, defect formation, and the integrity of the coating–substrate interface.

Of particular relevance, Yu et al. investigated the electroless deposition of ternary Ni–W–P coatings on Zr-4 and demonstrated that coating nucleation, growth, continuity, and corrosion behavior depend strongly on substrate pretreatment and deposition time [42]. However, Ni–W–P and binary Ni–P coatings represent chemically and structurally different systems. The incorporation of tungsten changes the plating-bath chemistry, involves induced co-deposition, and can modify the deposition kinetics, elemental and phase composition, and resulting physicochemical properties of the coating. Therefore, results obtained for Ni–W–P/Zr-4 cannot be directly transferred to the binary Ni–P/Zircaloy-4 system. Nevertheless, this work provides important evidence that electroless nickel-based coating formation on Zr-4 is strongly substrate-dependent and that appropriate surface activation and development of a continuous coating are essential. Systematic studies specifically addressing binary electroless Ni–P coatings on Zircaloy-4 remain scarce.

A similar knowledge gap exists for AM162H (AlBeMet). AM162H contains approximately 62 wt.% Be, with the balance consisting predominantly of aluminum, and combines low density, high specific stiffness, high thermal conductivity, and attractive irradiation-related properties [43]. To the best of our knowledge, peer-reviewed studies systematically investigating electroless Ni–P deposition directly on AM162H have not been identified in the available literature. The available direct information is mainly represented by industrial technical reports and conference or company presentations. Engineering tests performed for Boeing demonstrated the feasibility of high-phosphorus electroless nickel plating on AM162H, while also showing that reproducible coating adhesion depends critically on surface preparation, etching, and zincate treatment [44]. Electroless nickel has also been applied to AM162H substrates for precision optical components, where it provides an amorphous and machinable surface [45]. Related peer-reviewed studies on other beryllium-containing materials, such as Cu–Be C17200, confirm the feasibility of electroless Ni–P deposition on Be-containing substrates [46], while recent investigations continue to demonstrate the technological relevance of Ni–P-coated lightweight metal substrates for high-precision optical applications [47]. These systems, however, are chemically and structurally distinct from AM162H and therefore cannot be used to infer its specific coating behavior directly.

For UCN applications, successful coating deposition alone is insufficient. High coating continuity, uniform thickness, minimal through-thickness defects, and stable adhesion are particularly important because local uncoated regions may expose the underlying substrate and contribute to increased UCN wall losses. Previous investigations of coatings developed for UCN storage and reflection have demonstrated fractional uncoated surface areas on the order of $10^{-4}$–$10^{-5}$, illustrating the stringent requirements placed on coating continuity [48]. Consequently, the nucleation and growth of Ni–P layers, the effects of substrate-specific pretreatment and deposition conditions, and the resulting structural, compositional, mechanical, adhesive, and corrosion properties require dedicated investigation for Zircaloy-4 and AM162H.

Accordingly, the novelty of the present study does not lie in electroless Ni–P deposition itself, which is a well-established coating technology, but in the systematic comparative investigation of binary electroless Ni–P coating formation and properties on Zircaloy-4 and AM162H as unconventional structural materials of interest for UCN-source components, using AMg6 as a reference aluminum-based substrate.

In the present work, electroless nickel plating using sodium hypophosphite as a reducing agent was selected for the formation of Ni-P coatings [49,50]. This process belongs to electroless deposition methods and is based on the chemical reduction of nickel ions directly on the catalytically activated substrate surface, with simultaneous incorporation of phosphorus into the growing layer. Unlike electrochemical deposition, electroless nickel plating does not require an external current source and enables coatings to be formed on surfaces of complex geometry with a more uniform thickness distribution. This is especially important for processing the internal surfaces of UCN source components. In addition, the composition and thickness of the Ni-P layer can be controlled by selecting the solution composition, temperature, pH, and deposition time. This makes the method convenient for comparative studies of coating formation on aluminum–magnesium, zirconium, and aluminum–beryllium substrates [51].

An additional reason for selecting electrolessly deposited Ni-P coatings is their expected suitability for operation under intense neutron-field conditions. Since the UCN source chamber will be located close to the reactor core, the coating must preserve its integrity, adhesion to the substrate, and functional properties under long-term exposure to neutron and gamma radiation. In practical terms, this means that the coating must provide radiation resistance at an accumulated neutron fluence on the order of 10^20^ n/cm^2^ over an estimated service lifetime of approximately 10 years. The dense structure of the nickel-phosphorus layer, its chemical resistance, and the possibility of obtaining a nonmagnetic state make such coatings promising for further testing under conditions close to those of a reactor-based UCN source [36,37]. At the same time, the issues of long-term radiation resistance, changes in coating adhesion to the substrate, and preservation of neutron-optical properties after irradiation require separate investigation.

The subject of the present work is the electroless deposition of nickel-phosphorus coatings on the surfaces of three materials of interest for UCN source components: the AMg6 aluminum–magnesium alloy, Zircaloy-4, and the AM162H aluminum–beryllium alloy, commercially known as AlBeMet. The AMg6 aluminum–magnesium alloy is considered as a comparative substrate for which nickel-plating technologies are generally better established. Zircaloy-4 and AM162H were selected as promising materials for reactor and cryogenic assemblies of a UCN source; however, information on the formation of electrolessly deposited Ni-P layers on these materials remains insufficient. Therefore, the main objective of this work is to experimentally establish the possibility of obtaining nickel-phosphorus coatings on these substrates and to identify the features of their formation depending on the nature of the substrate material.

This work demonstrates that electrolessly deposited nickel-phosphorus coatings can be formed not only on the AMg6 aluminum–magnesium alloy, but also on Zircaloy-4 and AM162H. At the same time, it is established that the deposition regime and solution composition strongly depend on the nature of the substrate. The solution that provides coating formation on AMg6 and Zircaloy-4 is not fully suitable for AM162H, indicating the need for a separate selection of the chemical regime for aluminum–beryllium alloys. This circumstance is of practical importance, since it shows that the choice of coating for UCN source components must take into account not only the required neutron-optical properties of the layer, but also the chemical state of the surface of the specific substrate.

A comprehensive characterization program was applied to the deposited coatings. Scanning electron microscopy (SEM), energy-dispersive X-ray spectroscopy (EDS), inductively coupled plasma optical emission spectrometry (ICP-OES), and X-ray diffraction (XRD) were used to examine the surface morphology, coating thickness, elemental composition, elemental distribution, and phase-structural state. In particular, EDS provided spatially resolved information on the elemental composition and distribution within selected surface regions, whereas ICP-OES was employed as an independent quantitative technique for determining the elemental composition of the investigated coatings. These investigations were complemented by surface profilometry and instrumented nanoindentation to determine the roughness parameters, hardness, elastic modulus, and indentation stiffness of the coatings. Their adhesion behavior was qualitatively assessed by scratch testing followed by SEM examination of the resulting damage, while corrosion resistance was evaluated by electrochemical polarization measurements in an aqueous NaCl environment followed by Tafel analysis. Together, these complementary techniques provide a comprehensive materials-science characterization of the deposited Ni–P coatings and enable their structural, compositional, mechanical, surface, adhesive, and corrosion characteristics to be compared for the three investigated substrates.

Thus, the present work is aimed at experimentally substantiating the possibility of using electrolessly deposited nickel-phosphorus coatings on aluminum–magnesium, zirconium, and aluminum–beryllium substrates of interest for the UCN production. The obtained data expand the range of application of Ni-P coatings in UCN physics and show that, for each structural material, the conditions of electroless nickel plating must be selected individually, taking into account the substrate composition, surface state, and expected operating conditions in the UCN source.

## 2. Materials and Methods

### 2.1. Samples and Sample Preparation

In the present work, plate-type specimens of three structural alloys were used as substrates for the formation of nickel-phosphorus (Ni-P) coatings: the AMg6 aluminum–magnesium alloy, the AM162H aluminum–beryllium alloy, and the Zircaloy-4 zirconium alloy. The choice of these materials was determined by their potential applicability in components of neutron-physics facilities, including UCN storage and transport systems, in which materials must simultaneously meet requirements for low weight, sufficient mechanical strength, corrosion and radiation resistance, and technological compatibility with functional Ni-P coatings.

The AMg6 aluminum–magnesium alloy was used as the aluminum–magnesium substrate. This alloy belongs to wrought aluminum alloys of the Al-Mg-Mn system [52]. In terms of its chemical nature, this material corresponds to high-magnesium 5xxx-series aluminum alloys and can be described as an Al-6Mg-0.6Mn-type alloy. According to literature data for wrought aluminum alloy AMg6, the main alloying elements are magnesium, present in an amount of 5.8–6.8 wt.%, and manganese, present in an amount of 0.5–0.8 wt.%, while aluminum constitutes the alloy matrix. Chemical composition of AMg6 alloy is shown in Table 1. The density of the material is approximately 2.65 g/cm^3^. In the present work, this alloy was considered as a baseline lightweight aluminum structural material for a comparative assessment of the possibility of forming Ni-P coatings on substrates of different chemical nature.

The AM162H alloy, which belongs to the AlBeMet family of materials, was used as the aluminum–beryllium substrate. According to the manufacturer’s data, AM162H is an aluminum–beryllium material with a composition of Al-62 wt.% Be. Chemical composition of AM162H alloy is shown in Table 2. This material is characterized by a low density of approximately 2.10 g/cm^3^, a high elastic modulus of about 193 GPa, a Poisson’s ratio of 0.17, a linear thermal expansion coefficient of 13.9 ppm/°C at 25 °C, and a thermal conductivity of approximately 210 W/(m·K). The combination of low density and increased stiffness makes AM162H a promising structural material for applications in which mass, geometrical stability, and thermal conductivity are critical. The AM162H specimens were purchased from Materion Brush Inc.,Mayfield Heights, OH, USA. [53].

The Zircaloy-4 alloy [54], supplied in plate form by Qingdao XinHehui Steel Co., Ltd. (Qingdao, Shandong, China) [55], was used as the zirconium substrate. According to the supplier’s data, the chemical composition of the material includes zirconium as the matrix element, with alloying additions of tin, iron, and chromium: Sn, 1.5 wt.%; Fe, 0.2 wt.%; and Cr, 0.1 wt.% (see Table 3). The oxygen content does not exceed 0.10 wt.%. The use of Zircaloy-4 in the present work is motivated by the practical importance of zirconium alloys for nuclear-physics and reactor applications, where corrosion resistance, radiation stability, and compatibility with a neutron environment are essential.

Nickel plating of AMg6, AM162H, and Zircaloy-4 was carried out by electroless deposition using two different solutions. For AMg6 and Zircaloy-4, solution (I) was used, with the following composition: Ni(CH_3_COO)2·4H_2_O, 19 g/L; NaH_2_PO2·H_2_O, 15 g/L; and CH_3_COOH, 12 mL/L, with ρ = 1.05 g/cm^3^. The pH was 4.1-4.3. Deposition was performed at 85 °C, with a bath loading of 1 dm^2^/L and a deposition time of 3 h. For AM162H, solution (II) was used, with the following composition: NiSO4·4H_2_O, 20 g/L; NaH_2_PO2·H_2_O, 30 g/L; and Na_4_P_2_O_7_, 50 g/L. The pH was 9-10. Deposition was performed at 50 °C, with a bath loading of 1 dm^2^/L and a deposition time of 2 h.

The surface of AMg6 was pretreated as follows. First, the plates were cleaned with Vienna lime to remove residual mechanical contaminants from the specimen surface, rinsed, and then immersed for 2 min in a 25% NaOH solution at room temperature for surface degreasing. The specimens were subsequently treated in a HNO_3_ solution diluted 1:1 for 40 s to remove smut formed during the alkaline degreasing step. The plate surface was then chemically polished by immersion in a solution containing H_3_PO_4_, 1500 g/L, and HNO_3_, 60 g/L, at 70 °C for 1 min. After rinsing, immersion deposition of a preliminary Ni-P layer was carried out by holding the plate in a solution containing NiCl2·6H_2_O, 20 g/L, and H_3_PO_4_, 1420 g/L, for 45 s at 50 °C. The specimen was then rinsed and immersed again in the same solution for an additional 15 s at 50 °C. After this treatment, the specimen was rinsed and transferred to nickel-plating solution (I).

Surface preparation of AM162H is complicated by the high chemical activity of beryllium, since certain difficulties arise during nickel plating due to simultaneously occurring processes, namely beryllium etching and nickel deposition. Therefore, solution (II) was used for electroless nickel plating of this alloy. Prior to nickel plating, the AM162H specimens were immersed in a solution containing 25% NaOH and held at room temperature for 1 min to remove the aluminum oxide film. The specimens were then rinsed, transferred to solution (II), and held for 2 h.

The surface of Zircaloy-4 was first cleaned with Vienna lime, rinsed, and then immersed for 15 min in a solution containing 45 mL of concentrated HNO_3_, 10 mL of concentrated HF, and 45 mL of H_2_O in order to increase the surface wettability. The specimens were rinsed in running water, treated in an ultrasonic bath with a power of 50 W for 2 min, rinsed again in running water, and transferred to nickel-plating solution (I).

Heat treatment of the obtained coatings on all specimens was performed at 220 °C for 1.5 h. The post-deposition heat treatment was applied to improve the interfacial stability and adhesion of the coating.

### 2.2. Experimental Methods

The phase and structural state of the Ni-P coatings were investigated by X-ray diffraction (XRD) using MiniFlex 600 and PANalytical Empyrean diffractometers with Cu Kα radiation at room temperature. XRD measurements were performed in Bragg–Brentano geometry. Diffraction patterns were recorded over the 2θ range of 20°–120° with a step size of 0.026°.

Surface morphology and coating thickness were examined by scanning electron microscopy (SEM) using JCM-6000 Plus (JEOL, Tokyo, Japan), S-3400N (Hitachi, Tokyo, Japan), and TM4000Plus (Hitachi, Tokyo, Japan) microscopes. Secondary-electron (SE) and backscattered-electron (BSE) imaging modes were used where appropriate. Cross-sectional SEM images were used to determine the coating thickness.

The elemental composition of the coatings was characterized by energy-dispersive X-ray spectroscopy (EDS) with ZAF correction. EDS provided local information on the elemental composition and spatial distribution of the principal elements within selected coating regions. In addition, inductively coupled plasma optical emission spectrometry (ICP-OES) was used as an independent quantitative method for determining the elemental composition of the Ni–P coatings, particularly the Ni and P contents.

The mechanical properties and surface topography of the coatings were investigated using a NanoScan-4D (TISNCM, Troick, Russia) scanning nanohardness tester equipped with a Berkovich diamond indenter. Instrumented nanoindentation was performed at a maximum load of 50 mN with a holding time of 2 s. Hardness (*H*), elastic modulus (*E*), and indentation stiffness were determined from at least 10 indentations. Surface profilometry was performed using the same instrument, and the roughness parameters $R_{a}$, $R_{\mathrm{ms}}$, $R_{z}$ and the total height range were determined from the measured profiles.

Coating adhesion was evaluated qualitatively by scratch testing. Scratches were produced using the NanoScan-4D with a progressively increasing load up to 200 mN and, additionally, using a PMT-3 microhardness tester (LOMO, Saint Petersburg, Russia) equipped with a Vickers diamond indenter at a constant load of 2 N. The resulting scratch tracks were subsequently examined by SEM to assess cohesive and adhesive failure behavior.

The corrosion resistance of the coatings was evaluated electrochemically using a Gamry potentiostat/galvanostat in a 3 wt.% aqueous NaCl solution. Polarization curves were analyzed by Tafel extrapolation using Gamry Interface 1000E, Gamry Framework version 5 software to determine the corrosion current ($I_{\mathrm{corr}}$), corrosion potential ($E_{\mathrm{corr}}$), and corrosion rate ($V_{\mathrm{corr}}$).

## 3. Results and Discussion

Owing to the fact that systematic data specifically on electroless Ni–P coatings on AM162H and Zircaloy-4 remain limited, we started to examine the specific features of the electroless nickel-plating process on the AMg6 aluminum–magnesium alloy using solution (I). This approach made it possible to use AMg6 as a model substrate for the preliminary evaluation of the technological parameters of Ni-P coating deposition before proceeding to the nickel plating of beryllium- and zirconium-based alloys, namely AM162H and Zircaloy-4, respectively. The choice of solution (I) was determined by its relatively simple composition, technological reproducibility, and the possibility of flexible adjustment of the main process parameters. In particular, this solution makes it possible to regulate the buffering capacity by changing the concentration ratio of nickel ions to sodium hypophosphite within the range of 0.4–0.6, as well as to deliberately adjust the pH value and the content of the reducing agent, sodium hypophosphite. These features make solution (I) a convenient system for analyzing the regularities of electroless Ni-P deposition and for subsequently adapting the process to substrates of more complex chemical nature. In addition, this bath provides a high Ni-P coating deposition rate at a relatively low temperature of 85 °C, ensures stable long-term operation for 6–7 h, and remains stable during storage and use. Bath adjustment is performed once after 4 h of operation during a 6–7 h working cycle [56].

### 3.1. Ni–P Coating on AMg6 Alloy

**Figure 1:**
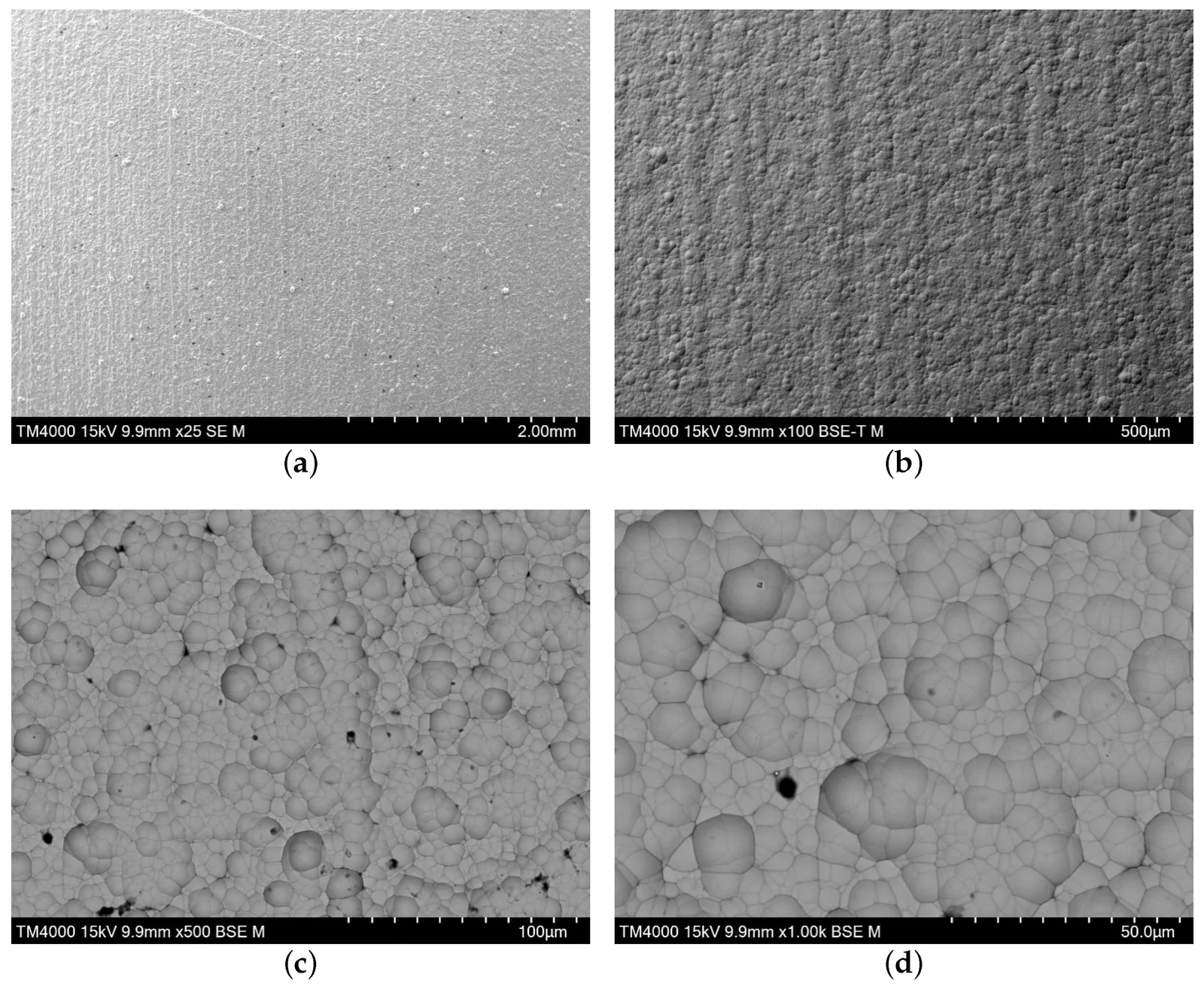
Surface morphology of the Ni–P/AMg6 coating at different magnifications: (**a**) BSE image (×25); (**b**) topographic BSE image (×100); (**c**) BSE image (×500); (**d**) BSE image (×1000).

**Figure 2:**
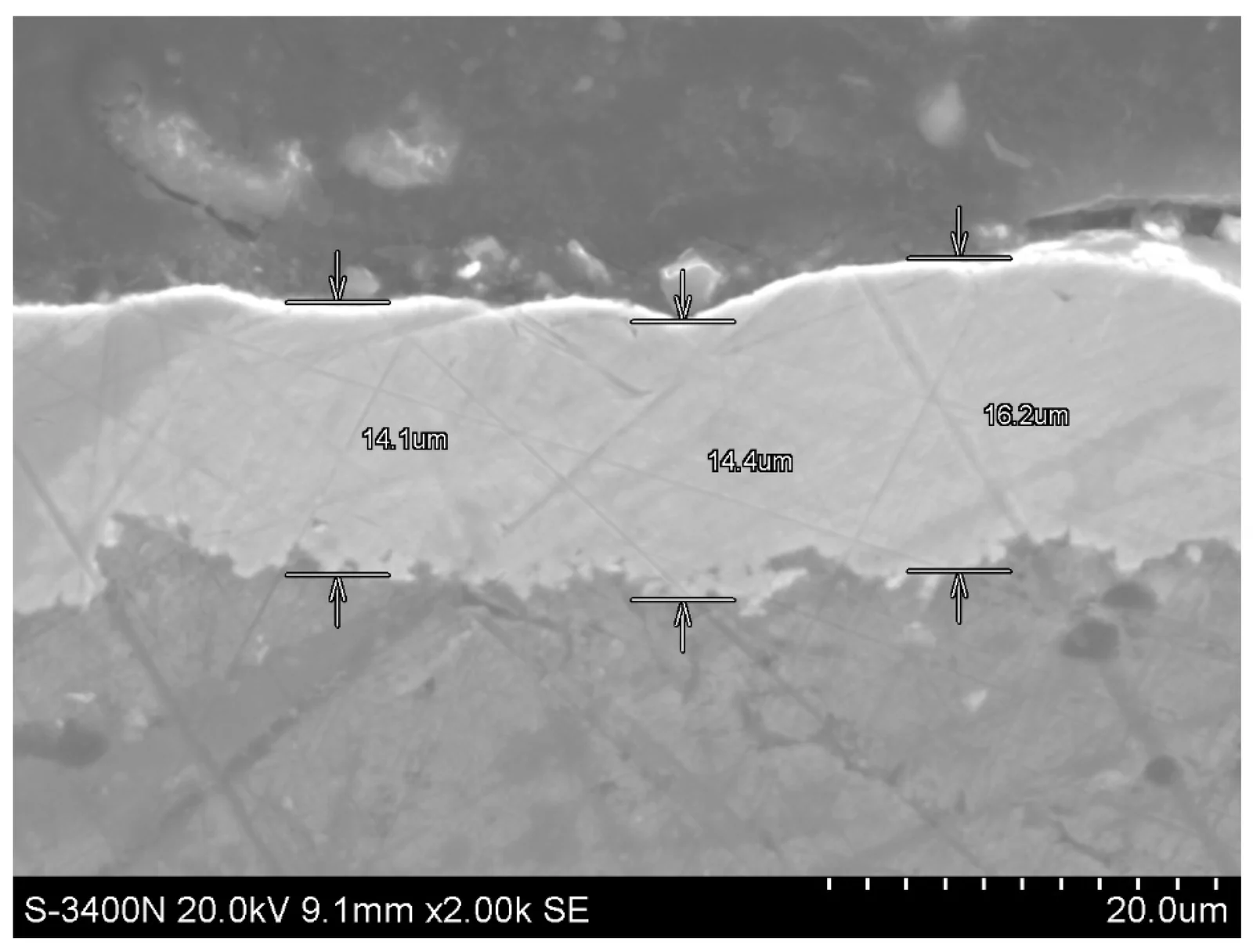
Cross -sectional thickness of the Ni–P/AMg6 coating.

**Figure 3:**
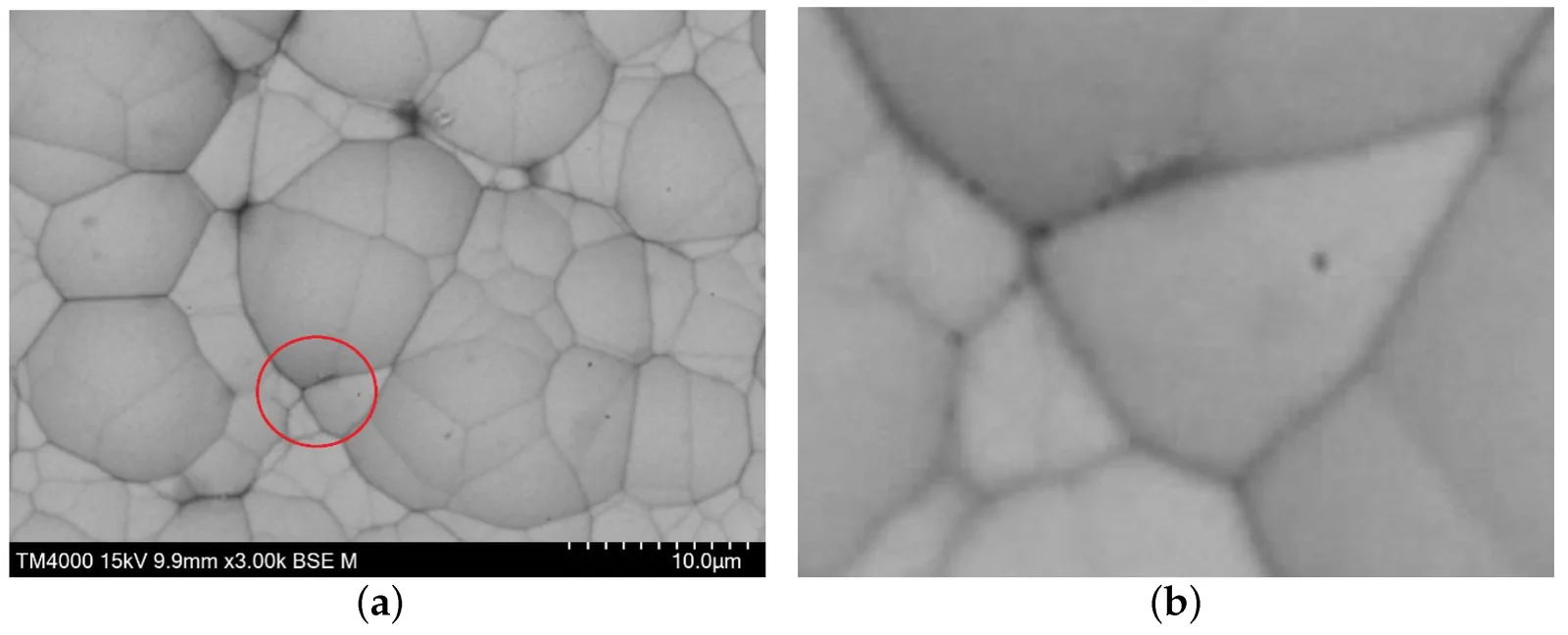
Surface morphology of the Ni–P/AMg6 coating at different magnifications: (**a**) (×100); (**b**) the enlarged highlighted surface area with pores.

A pretreated AMg6 specimen was immersed in solution (I) for electroless nickel plating. The specimen was held in the bath for a specified time, then rinsed, dried, and heat-treated. The Ni–P coating uniformly covers the substrate surface and conforms to its underlying topography (Figure 1a,b). At higher magnifications, the coating exhibits a characteristic nodular morphology composed of globular particles that form numerous surface agglomerates (Figure 1c,d). This morphology is consistent with an island-growth mechanism [57]. During the initial stage of deposition, particles nucleate at randomly distributed sites on the substrate surface. Subsequent lateral and vertical growth through the attachment of additional particles leads to the formation of agglomerates, which progressively coalesce into a continuous coating. Agglomerates formed during the early stages of deposition attain the largest dimensions, whereas subsequent islands develop within the remaining uncoated regions and partially on the surfaces of pre-existing agglomerates. Together, these structural features form a relatively uniform matrix from which larger, irregularly shaped agglomerates protrude. The globules range from 2 to 10 µm in size, while the agglomerates range from 10 to 30 µm. The coexistence of these two characteristic morphological features accounts for the observed variation in coating thickness from 14 to 16 µm (see Figure 2). At a limited number of isolated sites, pores with diameters of up to 300 nm are observed at the junctions between adjacent particles (Figure 3). Owing to their small size and low frequency of occurrence, these pores are considered to have a negligible effect on the mechanical properties and corrosion behavior of the coating.

**Figure 4:**
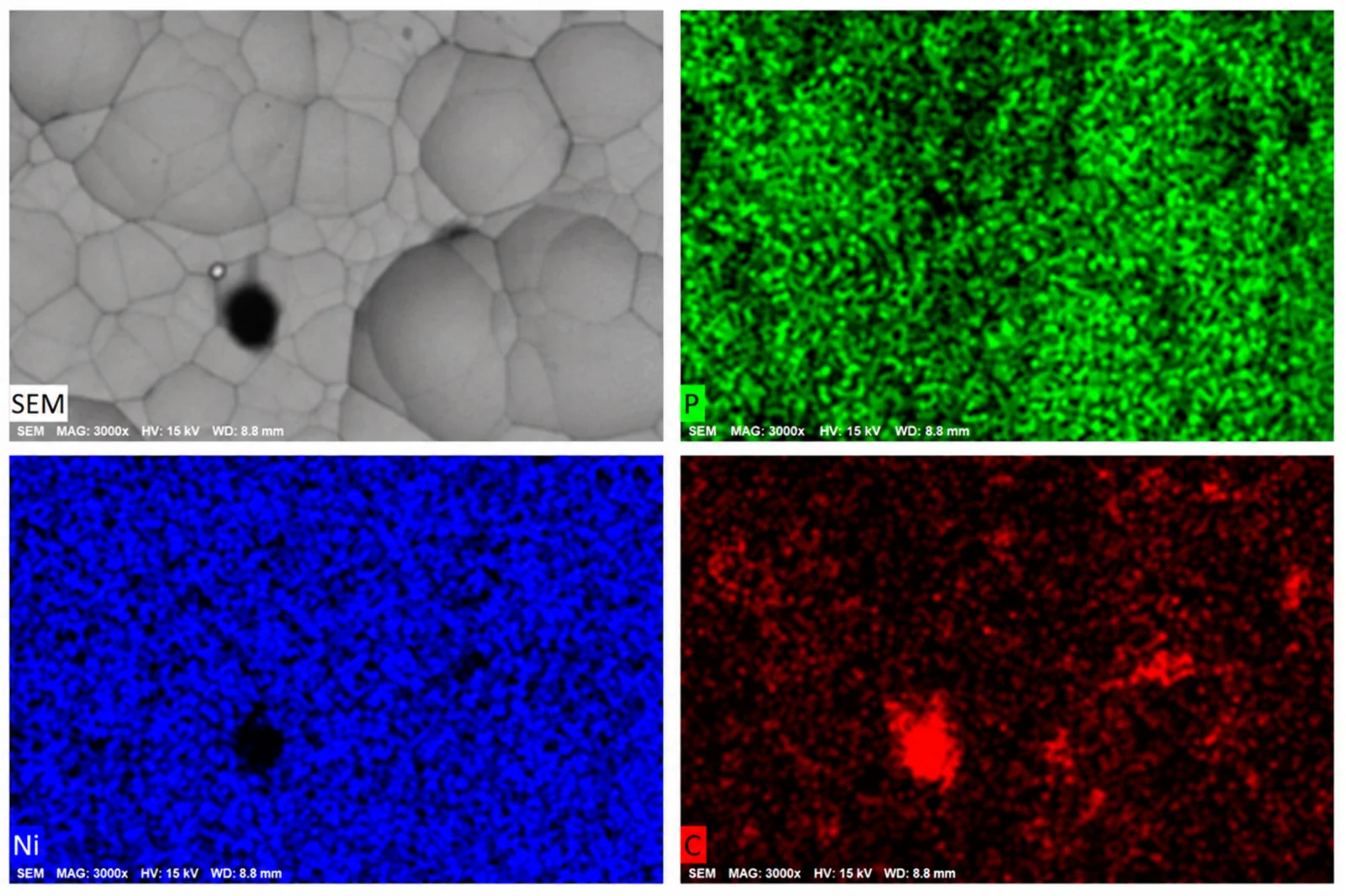
SEM and EDS images of the surface of the Ni–P/AMg6 coating. Elemental distribution maps: (C) carbon, (P) phosphorus, and (Ni) nickel.

The EDS microanalysis of the Ni-P coating deposited on the AMg6 specimen is presented in Figure 4. The colors in the elemental maps correspond to specific elements: Ni, P and C. A simultaneous analysis of the color contrast in the EDS distribution maps of Ni and P, as well as C, indicates a relatively uniform distribution of the detected elements over the coating surface.

According to the elemental analysis, the Ni-P coating deposited on the AMg6 substrate contained 81 wt.% Ni, 14 wt.% P, and 5 wt.% C. These values were determined with a relative uncertainty of approximately 2%. The traces of carbon detected in the Ni-P/AMg6 coating can be attributed to natural surface contamination of the substrate or coating. Based on the relative contents of nickel and phosphorus, the obtained coatings can be classified as high-phosphorus Ni-P coatings and are therefore expected to have a predominantly amorphous structure.

Thus, solution (I) can be used to obtain high-phosphorus, uniform Ni-P coatings.

**Figure 5:**
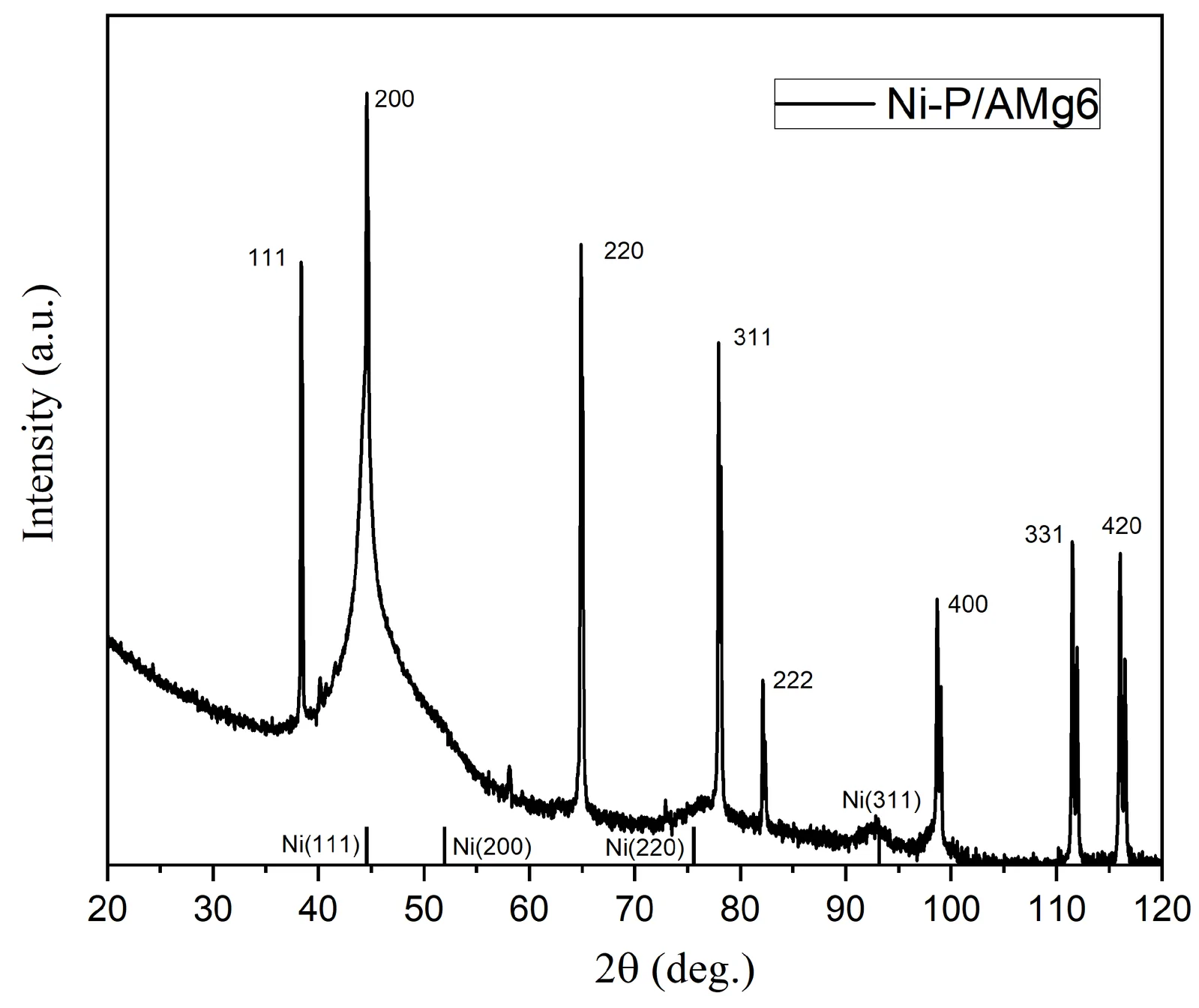
X-ray diffraction pattern of the Ni–P/AMg6 coating.

Figure 5 shows the X-ray diffraction pattern obtained from the specimen with a Ni–P layer deposited on the AMg6 aluminum–magnesium alloy substrate. Narrow and intense reflections attributed to the AMg6 aluminum alloy substrate are clearly observed in Figure 5, indicating the high crystallinity of the substrate. All registered reflections were indexed, and the corresponding indices are indicated in Figure 5. The crystalline reflections of the AMg6 substrate were indexed using the reference diffraction pattern for Al (Ref. Code 01-089-2837). Since the Ni–P coating deposited on the AMg6 surface is expected to be predominantly amorphous, this structural state should manifest itself in the diffraction pattern as a broad diffuse halo with intensity maxima located in the angular regions close to the characteristic diffraction maxima of crystalline nickel. The diffraction pattern exhibits broad diffraction maxima, whose positions correspond to the reflections of a nickel-based solid solution. In Figure 5, the positions of the nickel reflections with Miller indices (111), (200), (220), and (311) are marked by vertical ticks. Such peak broadening may be associated with the nanocrystalline state of the investigated phase. It should be noted that, despite the considerable coating thickness of 14–16 µm determined by SEM, diffraction maxima originating from the substrate are clearly observed in the diffraction pattern. The appreciable intensity of the substrate reflections may be attributed to the high defect density of the coating, accompanied by a reduction in its effective density. As a result, the linear attenuation coefficient of the X-ray radiation within the coating is reduced, allowing the radiation to penetrate to greater depths and, consequently, enabling the diffraction contribution from the substrate to be detected.

### 3.2. Ni–P Coating on AM162H Alloy

The next stage of the study was focused on the AM162H aluminum–beryllium alloy. For electroless nickel plating of this substrate, solution (II) was used, since in this case nickel deposition occurs simultaneously with beryllium etching.

**Figure 6:**
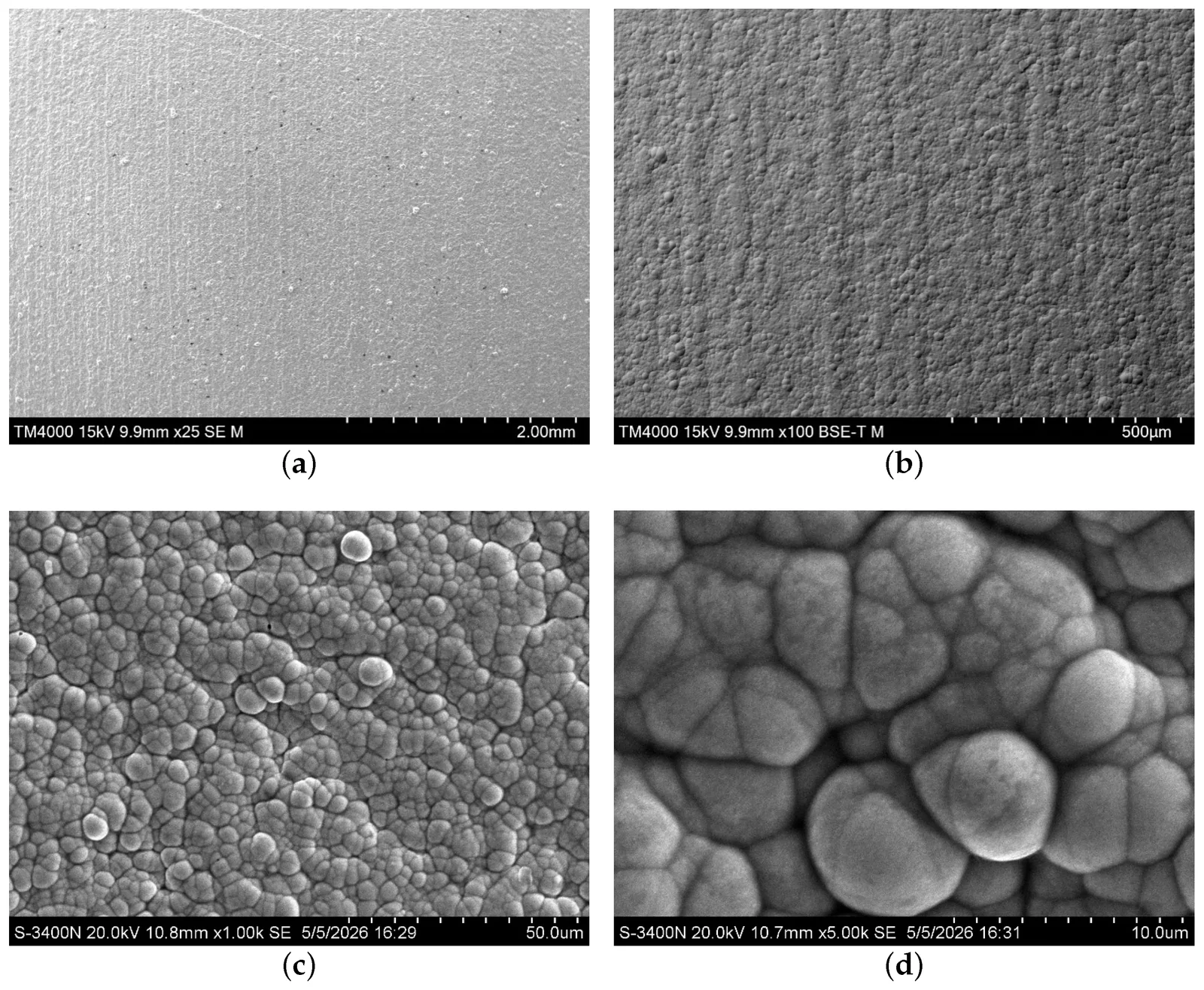
Surface morphology of the Ni–P/AM162H coating at different magnifications: (**a**) BSE image (×25); (**b**) topographic BSE image (×100); (**c**) SE image (×1000); (**d**) SE image (×5000).

**Figure 7:**
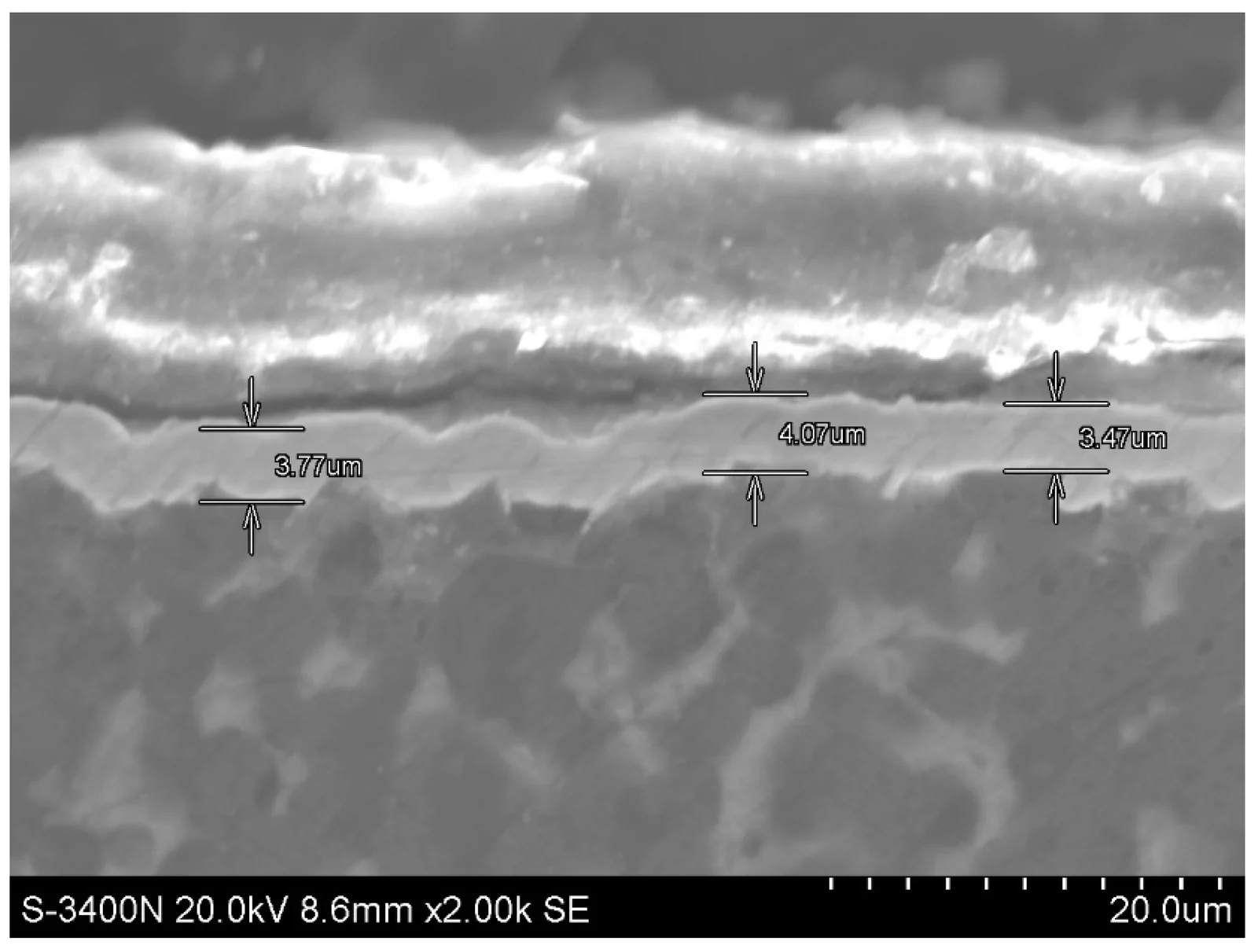
Cross-sectional thickness of the Ni–P/AM162H coating.

**Figure 8:**
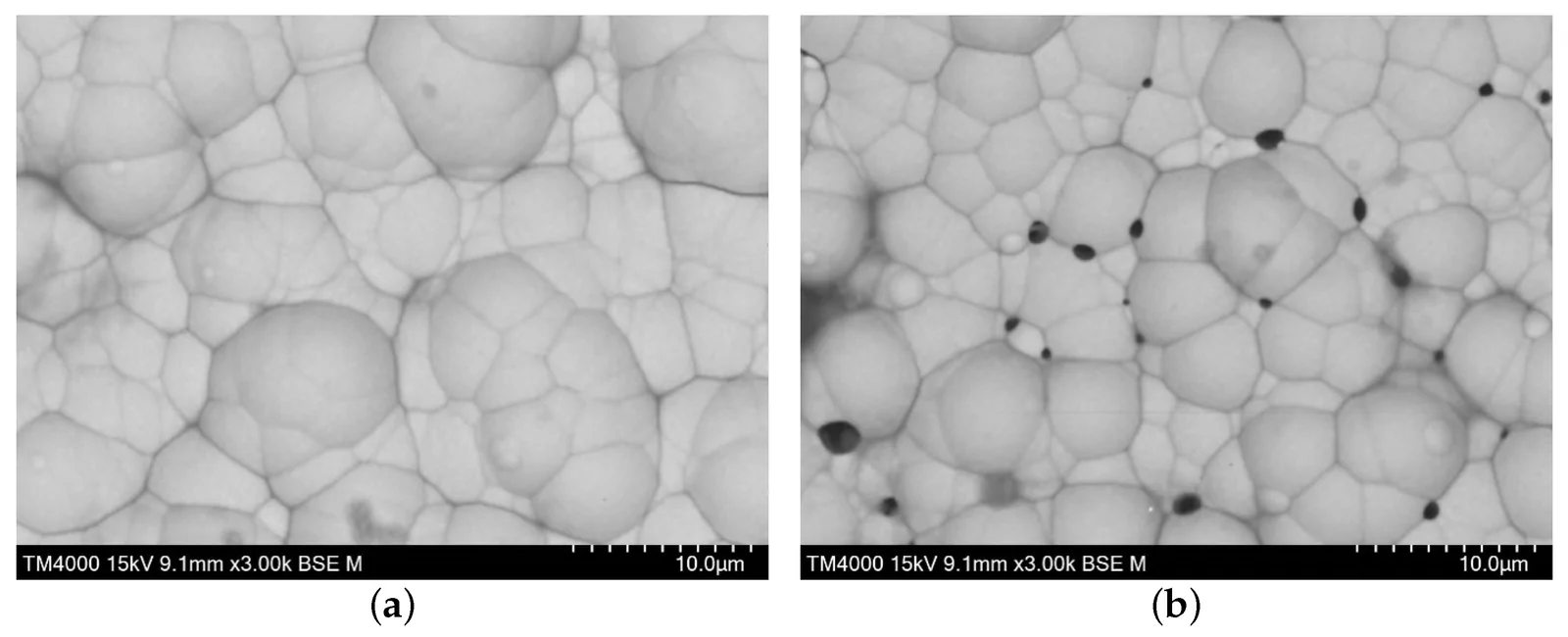
Surface of the Ni–P/AM162H coating: surface areas without pores (**a**) and with pores (**b**).

The Ni–P coating also uniformly covers the substrate and conforms to its surface topography (Figure 6a,b). The coating is composed of highly spherical globular particles and contains fewer and smaller agglomerates than the Ni–P/AMg6 coating (Figure 6c,d). The observed morphology suggests that, during the initial stage of deposition, the nucleation of discrete islands was accompanied by comparatively uniform coating growth over the entire substrate surface, thereby limiting the formation of large agglomerates. Consequently, the resulting coating consists predominantly of a layer of globules and small agglomerates, with relatively few large agglomerates protruding above the surface. The globules range from 1–7 µm in size, whereas the agglomerates range from 10–20 µm. Comparison of the dimensions of these structural features with the coating thickness 3–4 µm (see Figure 7) suggests that the agglomerates grow predominantly in the lateral direction. The spatial density of the boundaries between agglomerates and individual globules varies across the coating surface. Both pore-free regions (Figure 8a) and regions containing clusters of relatively large pores, approximately 1 µm in diameter (Figure 8b), were observed. These pores are located predominantly at the junctions between individual particles that are not incorporated into agglomerates.

**Figure 9:**
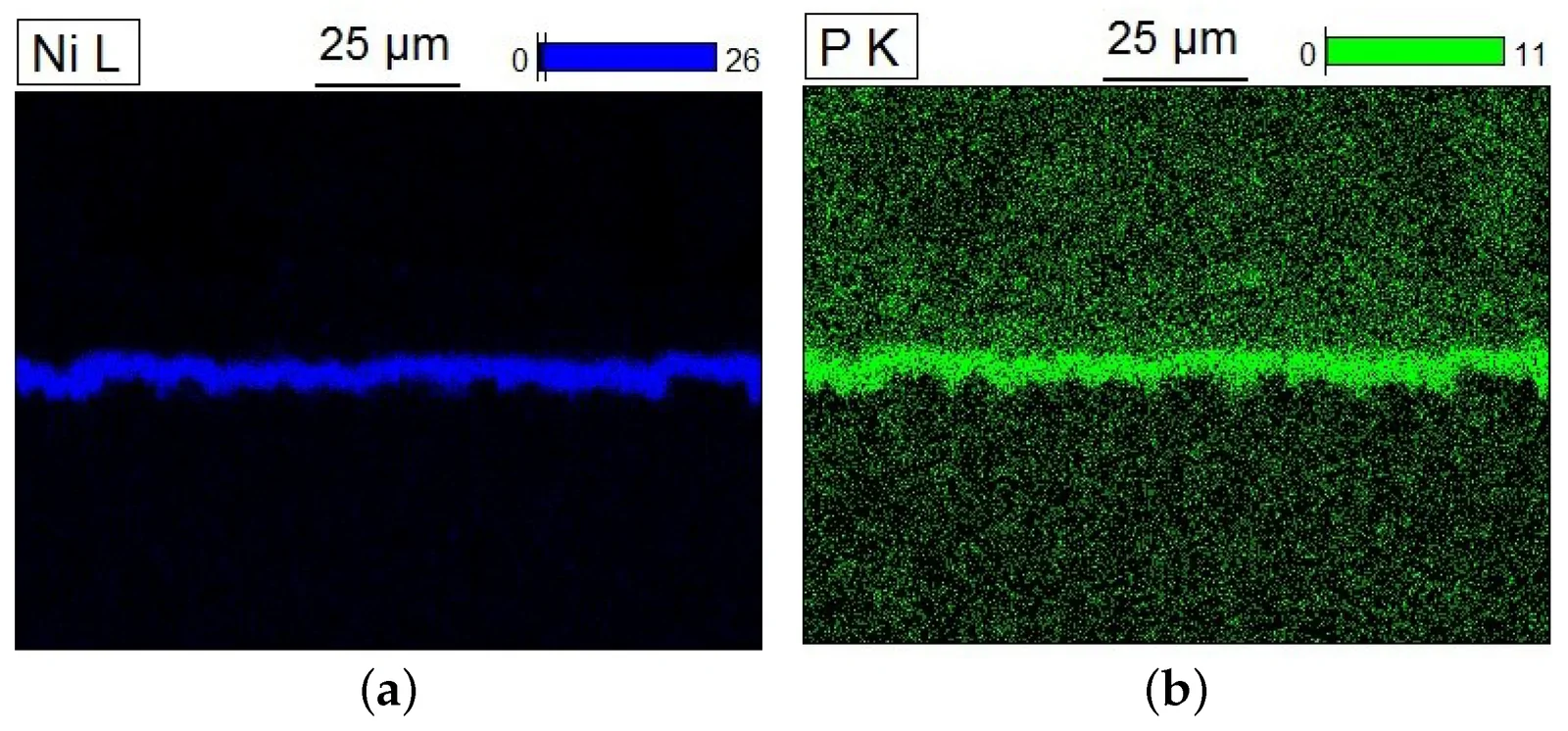
EDS elemental maps of the cross-section of the Ni–P coating on the AM162H alloy, showing the elemental distribution across the coating thickness: (**a**) nickel (Ni); (**b**) phosphorus (P).

**Figure 10:**
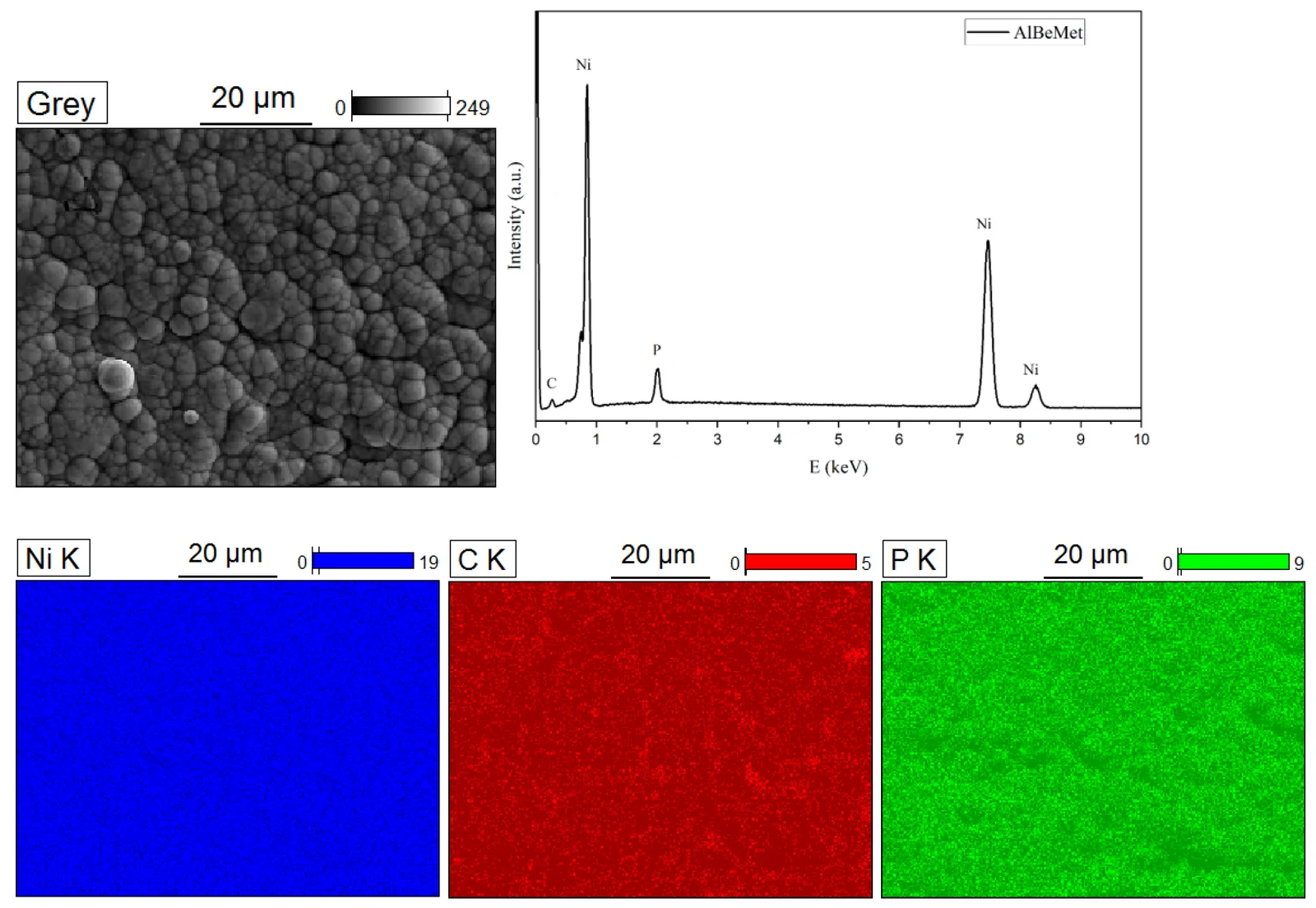
Elemental distribution maps of the Ni–P/AM162H coating.

Surface EDS analysis and cross-sectional EDS measurements, presented in Figure 9 and Figure 10 and Table 4, showed that phosphorus was distributed relatively uniformly both over the coating surface and throughout its thickness, with no pronounced phosphorus concentration gradient. Thus, the surface and cross-sectional EDS measurements, together with the independent ICP-OES analysis, consistently demonstrate that the Ni–P coating deposited on AM162H is a low-phosphorus coating [50]. The phosphorus content indicates that the coating may retain ferromagnetic properties, as reported in the literature for low-phosphorus Ni–P coatings [37]. This characteristic may limit its applicability in experiments involving polarized UCN. Achieving a nonmagnetic state would therefore require increasing the phosphorus content to above 10 wt.%, primarily through optimization of the composition and operating parameters of solution (II). The possible use of post-deposition heat treatment should be investigated separately because its effect on the phase structure and magnetic properties of Ni–P coatings depends strongly on the applied temperature–time regime.

**Figure 11:**
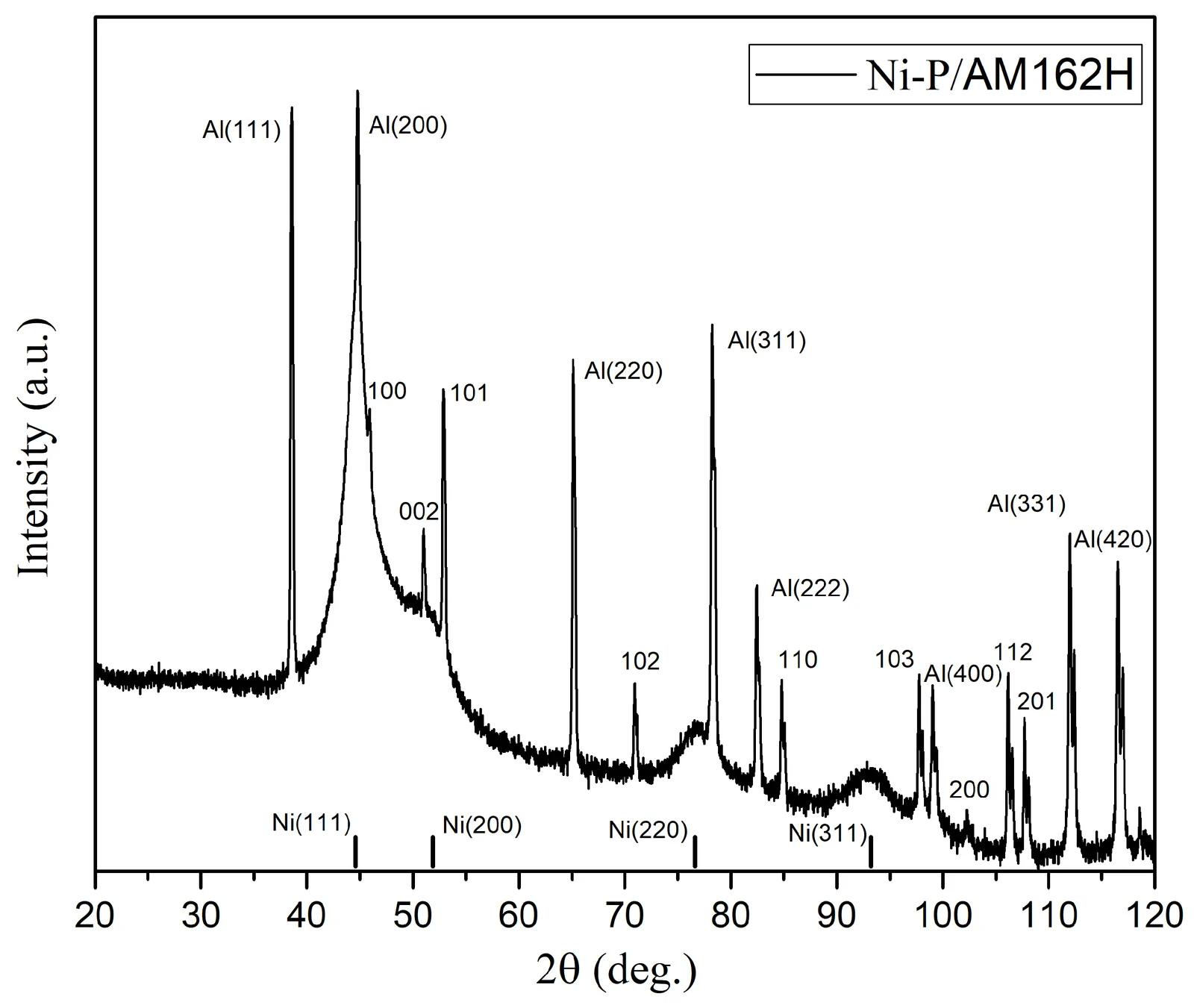
X-ray diffraction patterns of the Ni–P/AM162H coating.

Figure 11 shows the XRD pattern obtained from the Ni–P/AM162H specimen after deposition of the Ni–P layer on the crystalline AM162H substrate. As can be seen from Figure 11, the diffraction pattern contains numerous narrow and high-intensity reflections, which correspond to aluminum and beryllium, as indicated in Figure 11. The substrate reflections were indexed using the reference diffraction patterns for Be (Ref. Code 00-022-0111) and Al (Ref. Code 01-071-4623). In addition, the positions of the first four most intense nickel reflections are marked in the figure. As in the case of the Ni–P/AMg6 specimen, broad peaks associated with the Ni–P layer is observed in Figure 11. As in the previous case, the shape of the peaks may indicate nanoscale coherent scattering regions of the nickel-based solid solution. It should be noted that, despite the substantially smaller coating thickness of approximately 3–4 µm compared with the Ni–P layer deposited on the AMg6 substrate, the intensity of the diffuse scattering from the Ni–P/AM162H specimen is significantly higher. This difference indicates that the diffracted intensity from the amorphous Ni–P layer is determined not only by the geometric thickness of the coating, but also by its effective scattering volume and compactness. At the same time, as in the diffraction pattern of the Ni–P/AMg6 specimen, the structural reflections from the AM162H substrate remain clearly pronounced and are not completely suppressed by attenuation of the X-ray beam in the Ni–P layer. In the present case, this behavior is primarily associated with the small thickness of the coating. Since the Ni–P layer on AM162H is only about 3–4 µm thick, the incident and diffracted X-ray beams can partially penetrate through the amorphous coating and interact with the underlying crystalline substrate. As a result, the diffraction pattern contains both the broad diffuse contribution from the Ni–P layer and intense Bragg reflections from the AM162H substrate. Overall, the obtained XRD results demonstrate the formation of an amorphous Ni–P coating on AM162H using solution (II). The relatively high intensity of the diffuse halo, despite the small coating thickness, suggests that this deposition regime provides a comparatively dense and compact Ni–P layer on the aluminum–beryllium substrate. This result is important for the development of electroless nickel plating of beryllium-containing structural materials intended for UCN source components, including AM162H, commercially known as AlBeMet.

### 3.3. Ni–P Coating on Zircaloy-4 Alloy

**Figure 12:**
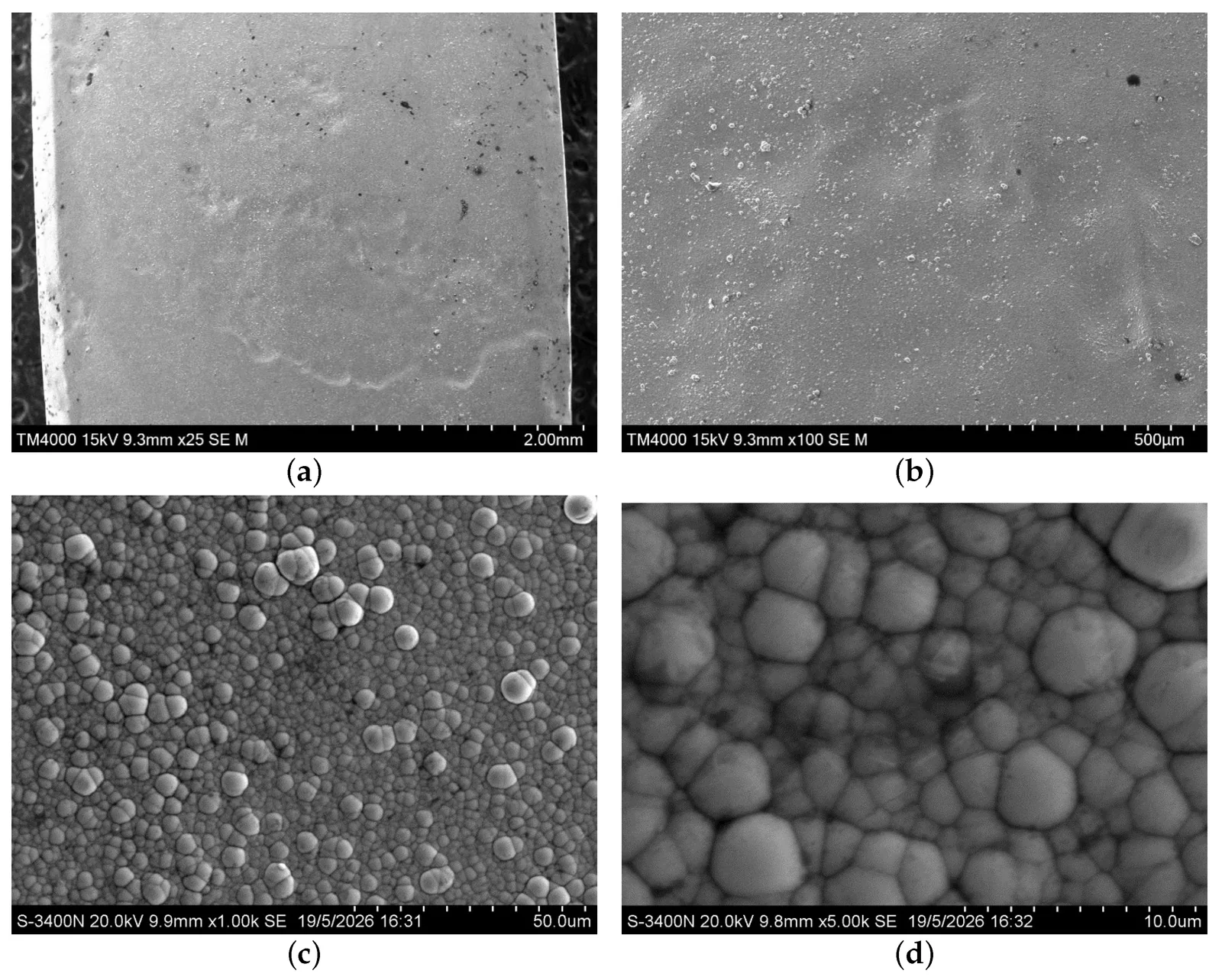
Surface morphology of the Ni–P/Zircaloy-4 coating at different magnifications: (**a**) BSE image (×25); (**b**) topographic BSE image (×100); (**c**) SE image (×1000); (**d**) SE image (×5000).

**Figure 13:**
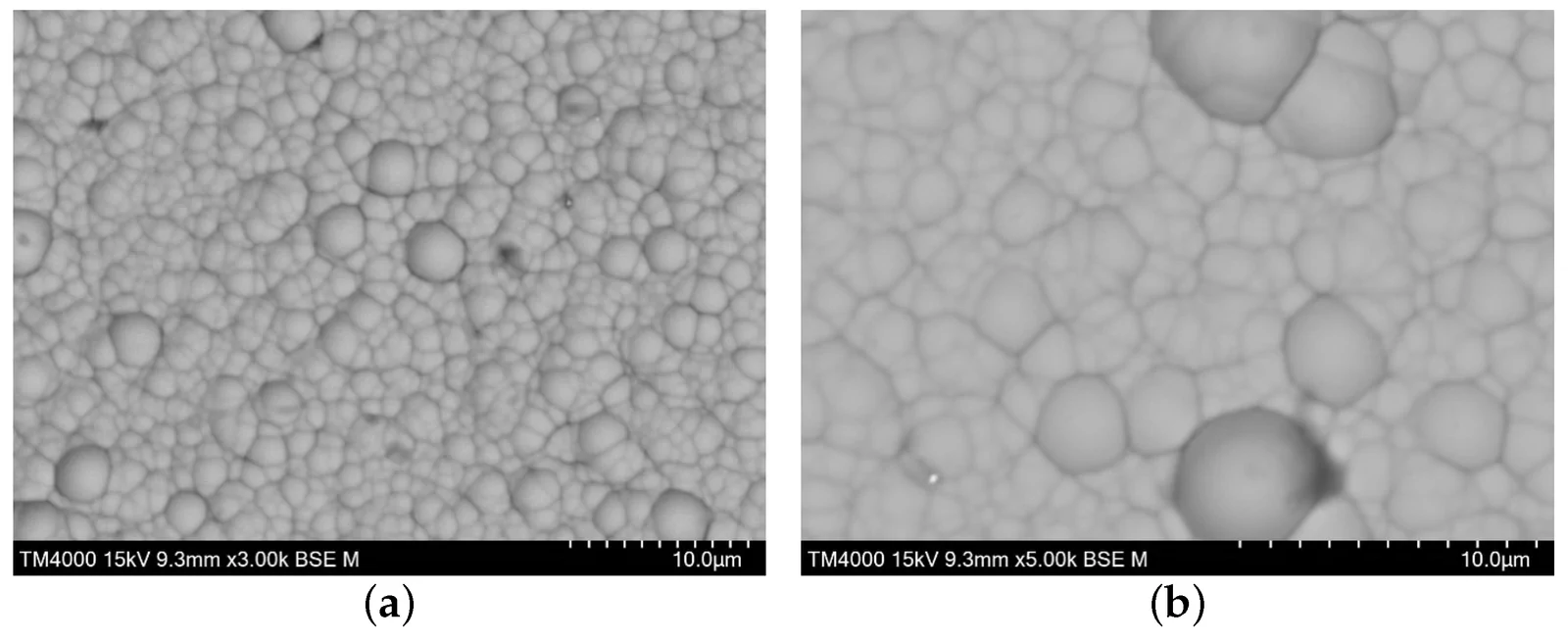
Surface of the Ni-P/Zircaloy-4 coating at various magnifications: (**a**) BSE image (×3000); (**b**) topographic BSE image (×5000).

**Figure 14:**
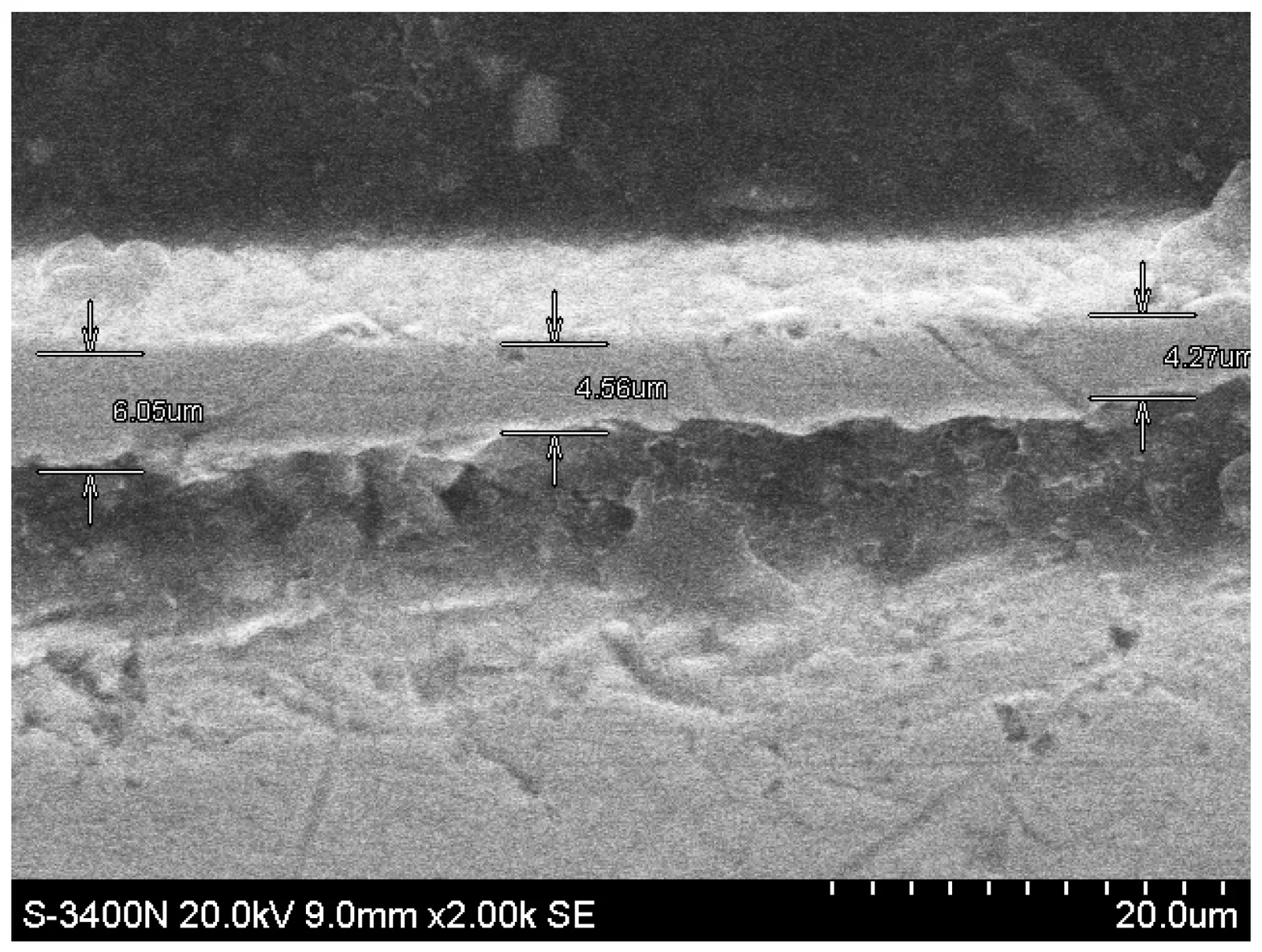
Cross -sectional thickness of the Ni–P/Zircaloy-4 coating.

Electroless nickel plating of the Zircaloy-4 alloy was carried out with solution (I) on a pretreated surface. The Ni–P coating likewise exhibits a relatively uniform distribution over the substrate and closely follows its surface topography (Figure 12a,b). The spherical globules are distributed more uniformly than in the coatings described above, with only a minimal number of agglomerates being present (Figure 12c,d). The globules range from 1–5 µm in size, whereas the agglomerates range from 5–8 µm. No pores were detected within the examined surface regions, and the globular particles exhibited a more compact arrangement (see Figure 13). Considering the coating thickness of 4–6 µm (see Figure 14) in relation to the globule dimensions, the coating can be assumed to consist predominantly of no more than one globular layer through its thickness. The observed morphology suggests that pronounced island-type growth was not the dominant mechanism of coating formation. Instead, nucleation and subsequent growth likely proceeded relatively uniformly and concurrently across the substrate surface, resulting in a highly compact coating structure.

**Figure 15:**
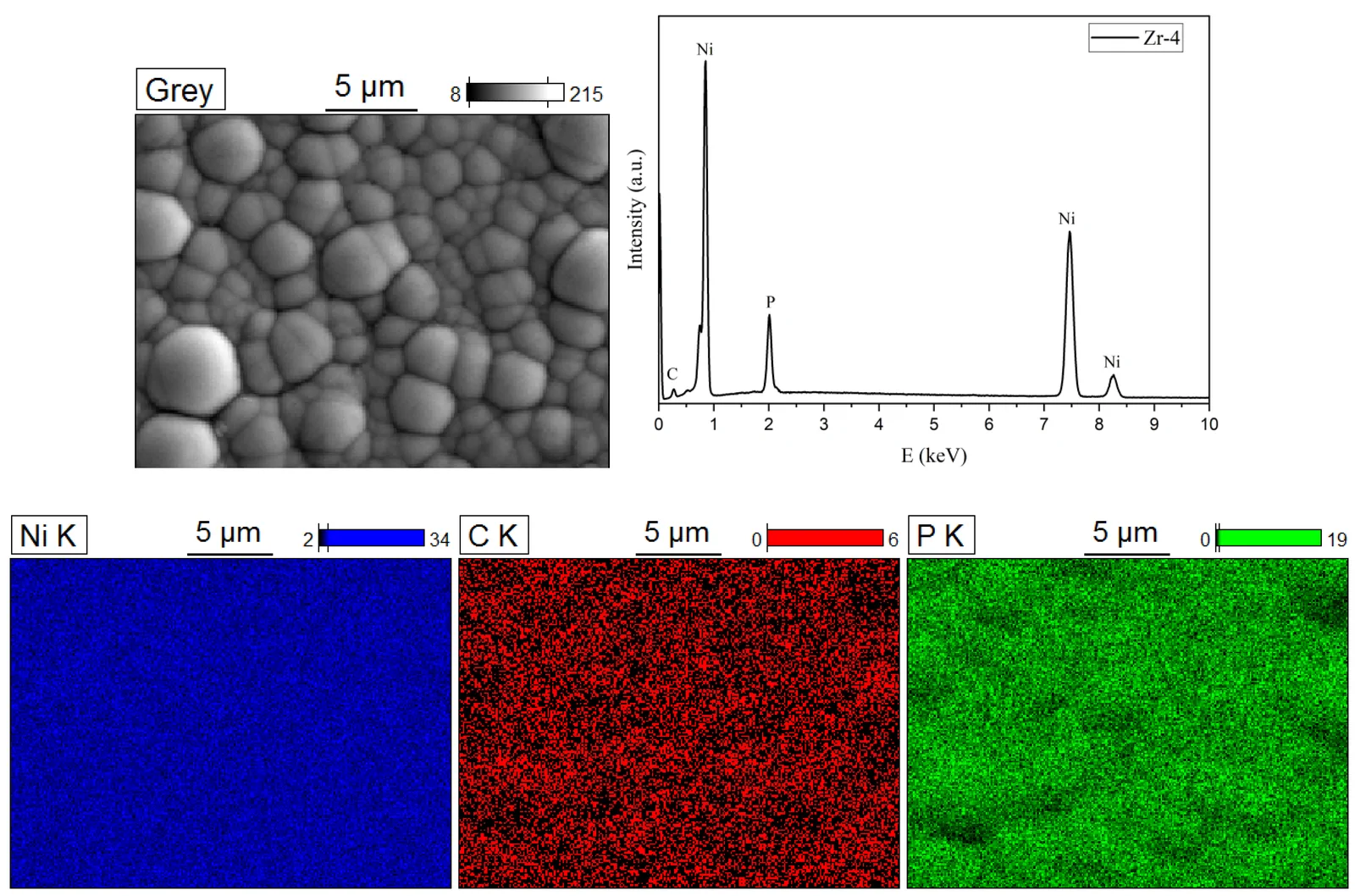
Elemental distribution maps of the Ni–P/Zircaloy-4 coating.

The elemental analysis data for the Ni-P/Zircaloy-4 coating, presented in Table 5 and Figure 15, confirm the formation of a high-phosphorus coating on the Zircaloy-4 substrate. As in the previous cases, the coating is characterized by a granular/nodular morphology with nearly spherical surface features. The elemental mapping data show a uniform distribution of nickel and phosphorus over the coating surface. No visible defects were detected in the SEM images of the Ni–P/Zircaloy-4 coating, demonstrating a dense and pinhole-free surface morphology.

**Figure 16:**
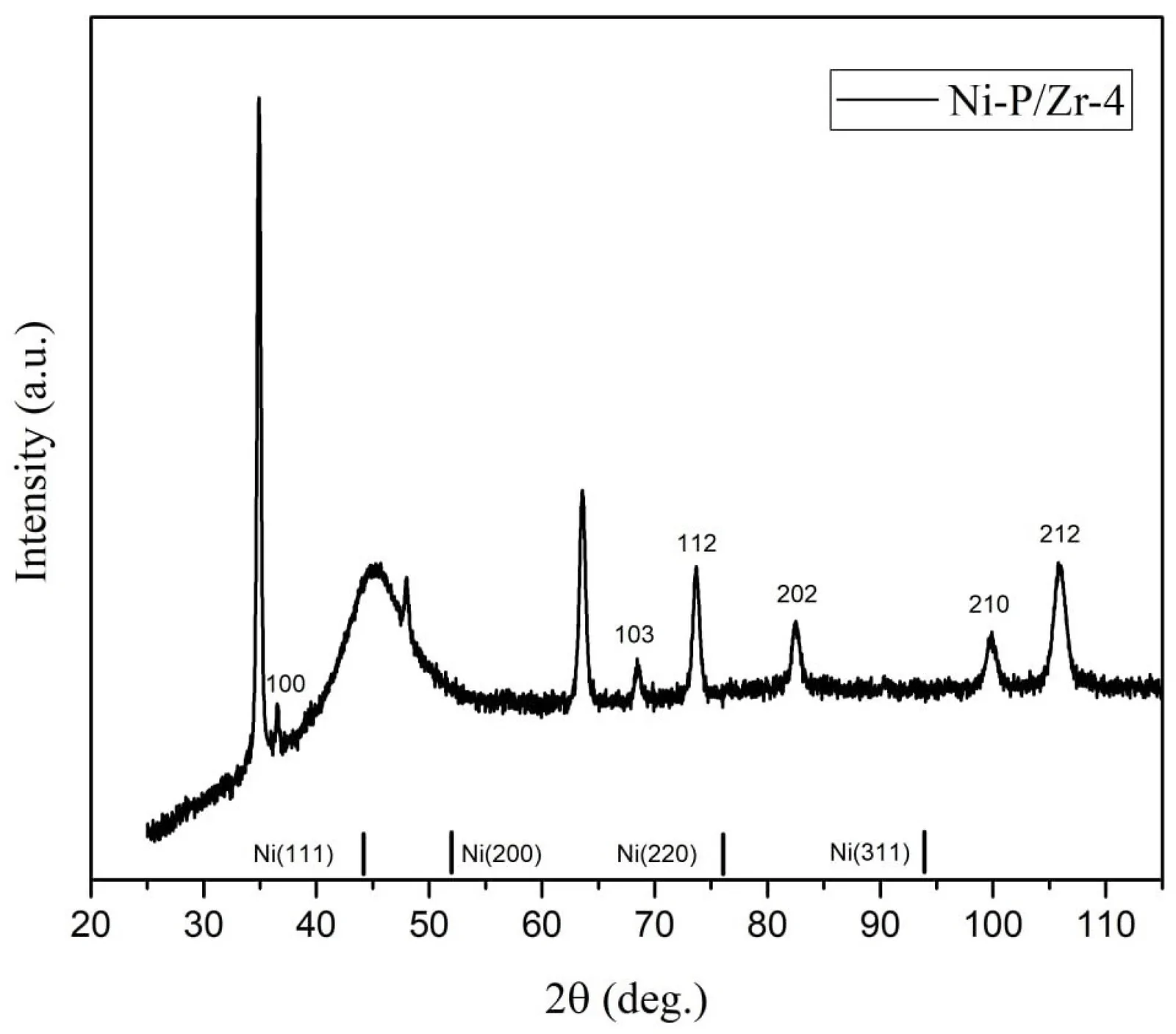
X-ray diffraction patterns of the Ni–P/Zircaloy-4 coating.

Figure 16 presents the XRD results for the Ni-P/Zircaloy-4 coating obtained in the present work. The crystalline reflections of the Zircaloy-4 substrate were indexed using the reference diffraction pattern for Zr (Ref. Code 01-089-3045). According to these data, the Ni-P coating is in an amorphous state. This is indicated by the presence of a broad, high-intensity diffuse peak with a maximum located at approximately 2θ = 42°.

### 3.4. Elemental Composition and Physicomechanical Characteristics of the Ni–P Coatings

To enable a direct comparison between the two analytical methods, the ICP-OES and EDS results were normalized to a total Ni + P content of 100 wt.% (Table 6). Carbon detected by EDS was excluded from the normalization. The normalized results obtained by the two analytical methods are in good agreement and establish the same sequence of phosphorus content.

The physicomechanical characteristics obtained by instrumented nanoindentation are summarized in Table 7. Ni–P/AMg6 showed the lowest mean hardness and elastic modulus, whereas the coatings on AM162H and Zircaloy-4 were substantially harder. The highest elastic modulus was measured for Ni–P/AM162H, while Ni–P/Zircaloy-4 exhibited the highest indentation stiffness. The comparatively large reported spreads in hardness and elastic modulus are consistent with local heterogeneity of the globular and nodular coating morphologies.

Comparison of the mechanical data with the ICP-OES results does not reveal a simple monotonic relationship between phosphorus content and mechanical response. Ni–P/AMg6 contains the highest phosphorus fraction but has the lowest hardness and elastic modulus, whereas Ni–P/AM162H combines the lowest phosphorus content with the highest values of both quantities. Ni–P/Zircaloy-4 has an intermediate phosphorus content, high hardness, and the highest measured indentation stiffness. The mechanical response must therefore be considered in relation not only to composition, but also to deposition conditions, coating morphology, thickness, and the underlying substrate.

**Figure 17:**
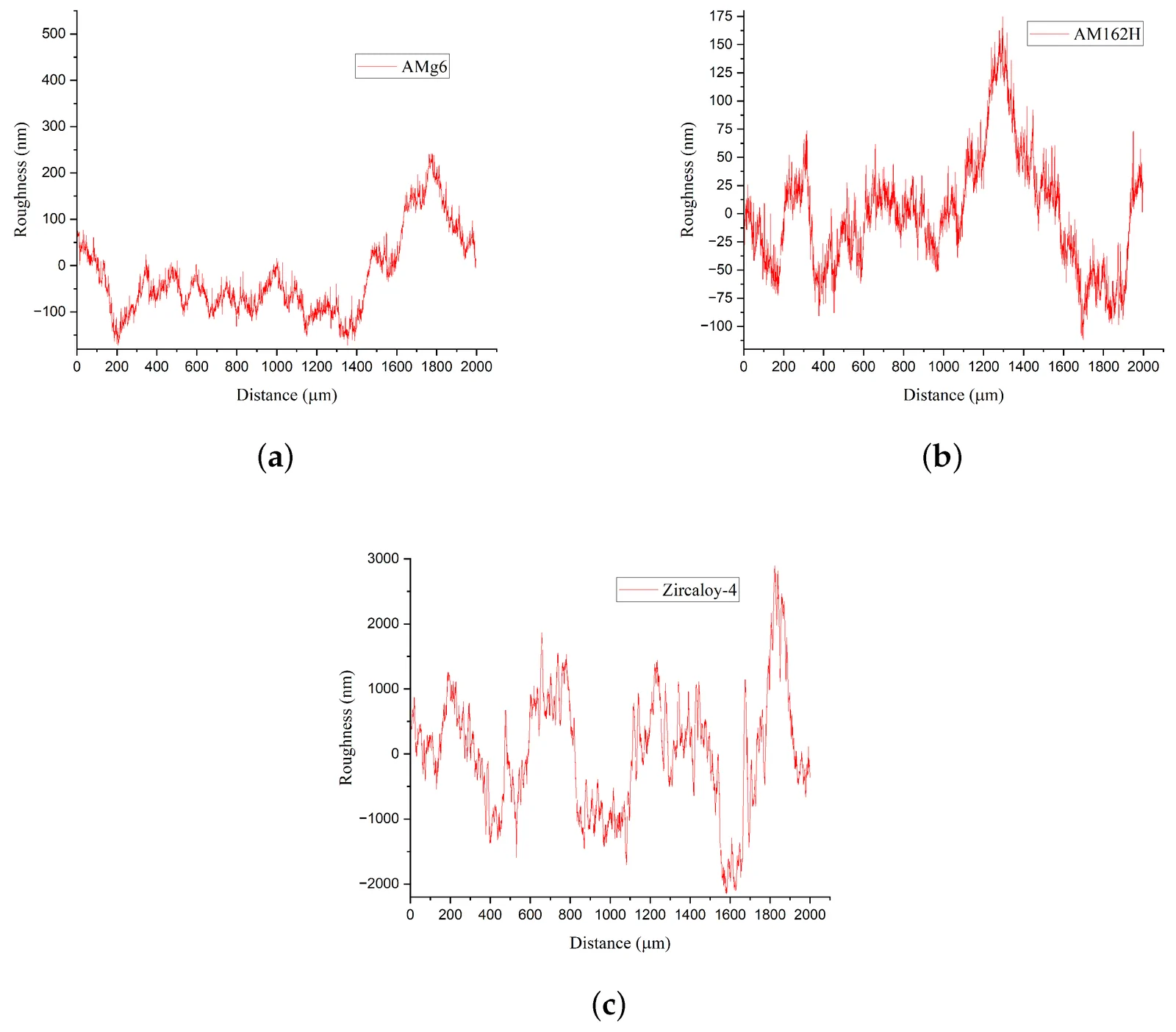
Representative surface profiles of the Ni–P coatings deposited on (**a**) AMg6, (**b**) AM162H, and (**c**) Zircaloy-4.

Surface profilometry revealed pronounced differences among the three systems (Figure 17 and Table 8). The arithmetic mean roughness increased from 94 nm for Ni–P/AMg6 to 130 nm for Ni–P/AM162H and 420 nm for Ni–P/Zircaloy-4. The root-mean-square roughness, ten-point profile height, and total measured height range followed the same ordering.

Despite its greater roughness amplitude, the Ni–P/Zircaloy-4 profile shows comparatively regular variations in peak height. This behavior agrees with the SEM observation of densely packed, relatively uniformly distributed spherical surface features. Its high roughness can therefore be associated with the height difference between adjacent globular features and their boundaries rather than with macroscopic discontinuities. The AMg6- and AM162H-based coatings display lower-amplitude but less regular profile variations. Differences among the three systems are consistent with their distinct surface pretreatments and coating-growth behavior.

**Figure 18:**
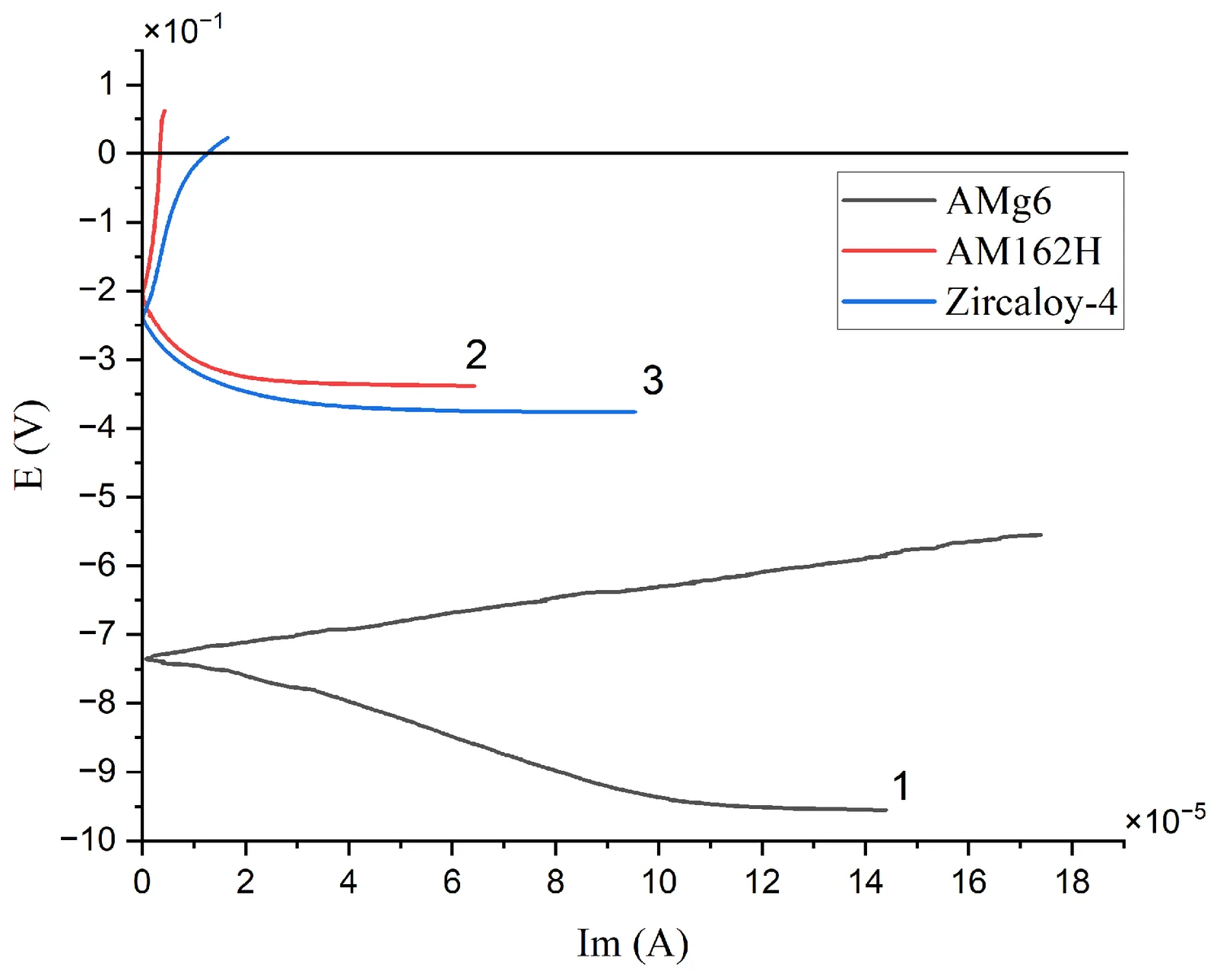
Tafel polarization curves for the Ni–P coatings on different substrates: 1—AMg6, 2—AM162H, and 3—Zircaloy-4.

The polarization curves used for the Tafel analysis are presented in Figure 18, and the derived corrosion parameters are listed in Table 9. The curve obtained for the Ni–P/AMg6 coating is shifted most strongly toward negative potentials and extends furthest toward higher current densities. In contrast, the Ni–P/AlBeMet coating exhibits the most positive corrosion potential. Although the Ni–P/AMg6 and Ni–P/Zircaloy-4 coatings differ considerably in their surface morphology and phosphorus content, their polarization curves demonstrate certain qualitative similarities in the evolution of the electrochemical response. Nevertheless, the quantitative corrosion parameters clearly distinguish the behavior of these coating systems.

Among the investigated coatings, Ni–P/AM162H exhibits the highest corrosion resistance. The corrosion characteristics of Ni–P/Zircaloy-4 are close to those obtained for Ni–P/AM162H, whereas Ni–P/AMg6 demonstrates substantially greater electrochemical corrosion activity. In particular, the corrosion current measured for Ni–P/AMg6 is approximately two orders of magnitude higher than the values obtained for the other two coatings. It should be noted, however, that the electrochemical corrosion measurements were conducted for only approximately 15 min and therefore primarily characterize the initial stage of the corrosion process. As reported in Ref. [58], prolonged exposure can promote the formation of a passivating layer on the surface of Ni–P coatings. Consequently, the corrosion resistance of these coatings may increase with exposure time as the protective surface layer develops.

Adhesion was assessed qualitatively by scratch testing. A progressively increasing load up to 200 mN did not penetrate the Ni–P/AM162H coating to a depth comparable with its full thickness and was therefore insufficient for evaluating interfacial failure. Additional tests were consequently performed at a constant load of 2 N [59]. Because this procedure does not yield a quantitative critical adhesion load, the results were interpreted comparatively from cracking, fragmentation, delamination, and coating separation observed by SEM.

**Figure 19:**
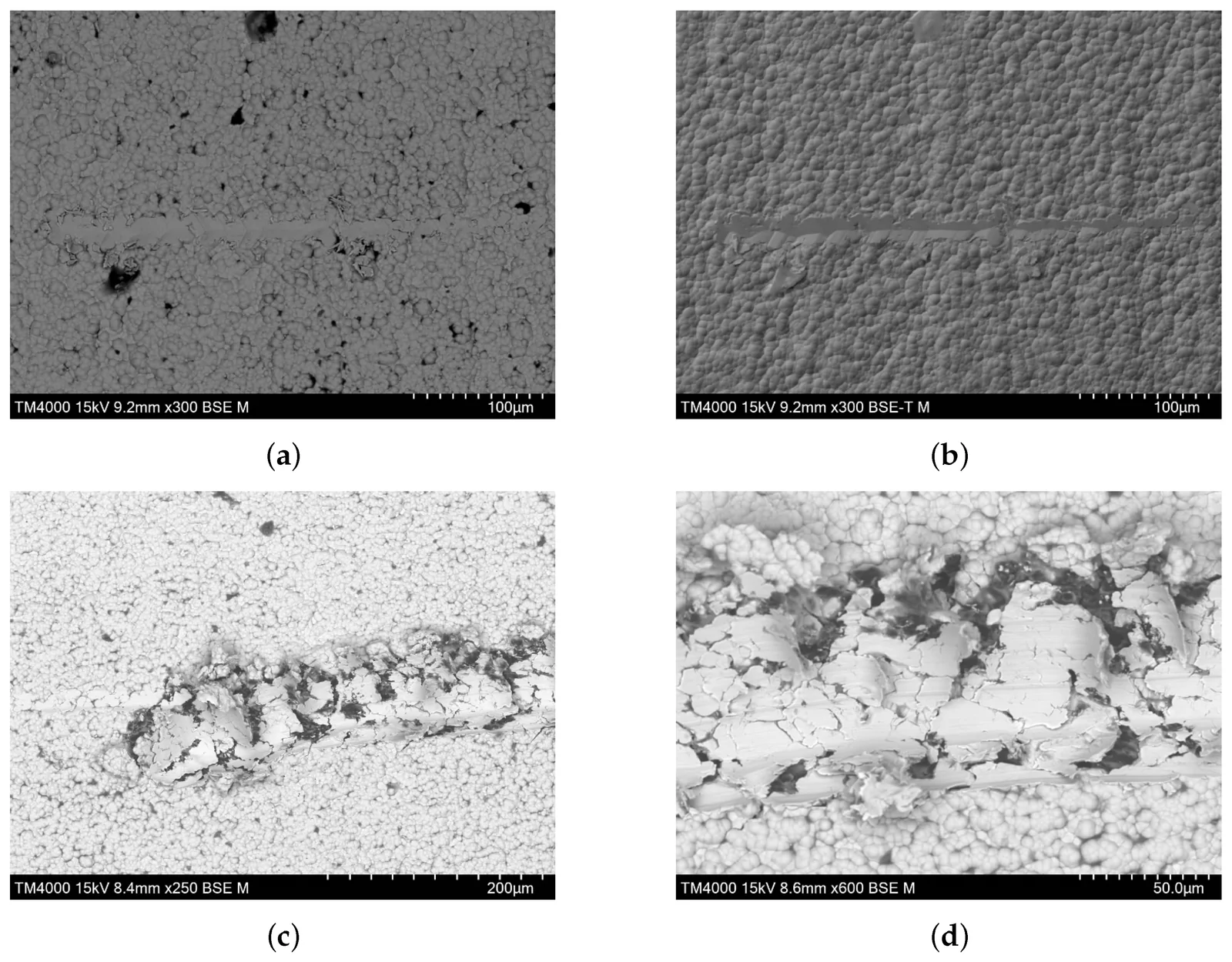
SEM images of scratch tracks on Ni–P/AM162H: (**a**) BSE and (**b**) topographic BSE images at ×300 after scratching with a progressively increasing load up to 200 mN; (**c**) BSE image at ×250 and (**d**) BSE image at ×600 after scratching at a constant load of 2 N.

**Figure 20:**
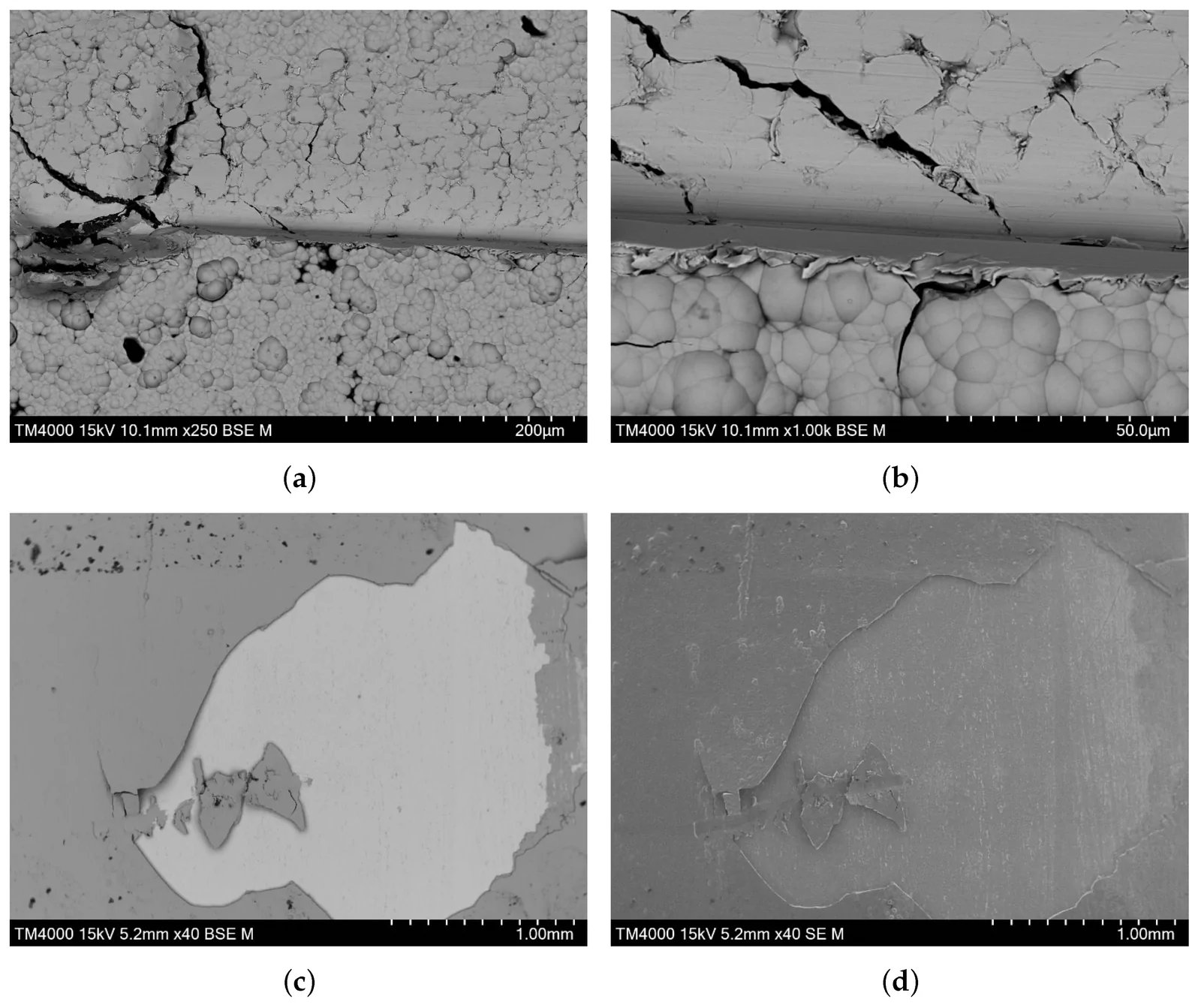
SEM images of scratch tracks produced at a constant load of 2 N: Ni–P/AMg6 in BSE mode at (**a**) ×250 and (**b**) ×1000; Ni–P/Zircaloy-4 at ×40 in (**c**) BSE and (**d**) SE modes.

For Ni–P/AM162H, damage developed progressively along the 2 N scratch track, but pronounced delamination from the substrate and brittle edge fracture were not observed (Figure 19c,d). This response indicates comparatively ductile deformation and satisfactory interfacial adhesion. Ni–P/AMg6 exhibited brittle cohesive cracking near the scratch while remaining comparatively well attached to the substrate (Figure 20a,b), likewise indicating satisfactory interfacial adhesion despite the more brittle local fracture mode.

In contrast, scratching of Ni–P/Zircaloy-4 caused detachment of a comparatively large coating fragment (Figure 20c,d). The size of the detached region indicates that the coating retained appreciable cohesive integrity, whereas separation occurred preferentially at or near the coating–substrate interface. Under the applied conditions, the interfacial adhesion of Ni P/Zircaloy-4 was therefore substantially weaker than that of the coatings deposited on AMg6 and AM162H. Accordingly, further studies are required to identify the factors limiting interfacial bonding and to improve the adhesion of the Ni–P coating to the Zircaloy-4 substrate. The importance of controlling the coating–substrate interface is also supported by recent studies of other functional coating systems. Ding et al. demonstrated that the interfacial adhesion of multilayer (Zr,Cr,Al)N coatings on an aluminum-alloy substrate depends strongly on the deposition conditions, coating microstructure, and interfacial architecture. In particular, the use of a dedicated metallic adhesion layer and optimization of the deposition parameters contributed to improved coating–substrate bonding and mechanical integrity [60]. Although the coating chemistry and deposition method differ from those used in the present work, these results emphasize the general importance of interfacial engineering for improving the adhesion and mechanical stability of functional coatings. Potential optimization strategies include refinement of the surface pretreatment and activation procedures, adjustment of the deposition parameters, and the introduction of a thin, chemically compatible metallic interlayer, such as aluminum, between the substrate and the Ni–P coating. The effectiveness of these approaches should be evaluated systematically, taking into account their influence on coating continuity, corrosion resistance, thermal stability, and the functional properties required for UCN applications.

Taken together, these measurements demonstrate that the three electroless Ni–P coatings possess distinct combinations of elemental composition, mechanical response, surface topography, corrosion resistance, and adhesion behavior. ICP-OES independently confirms the compositional trend established by EDS, while the physicomechanical results show that phosphorus content alone does not determine coating performance. Ni–P/AM162H combines high hardness and elastic modulus with the best corrosion resistance and satisfactory adhesion. Ni–P/Zircaloy-4 exhibits high hardness, the highest indentation stiffness, a regular dense morphology, and corrosion resistance close to that of Ni–P/AM162H, although its interfacial adhesion requires further optimization. Ni–P/AMg6 has lower hardness and elastic modulus and poorer electrochemical corrosion resistance, but it remains satisfactorily bonded to the substrate. These findings show that coating performance is governed by the combined effects of composition, growth morphology, surface preparation, and substrate material.

## 4. Conclusions

The feasibility of forming electroless Ni–P coatings on the AMg6 aluminum–magnesium alloy, the AM162H aluminum–beryllium alloy, and Zircaloy-4 was experimentally shown. The deposition conditions were found to depend strongly on the chemical nature and surface state of the substrate. The acidic solution (I) was suitable for AMg6 and Zircaloy-4, whereas AM162H required the alkaline solution (II) and a separate surface-preparation procedure. These results confirm that electroless nickel-plating conditions must be selected individually for each substrate.

SEM study showed that the coatings uniformly covered the substrates and generally followed their surface topography, although their thickness and growth morphology differed substantially. The Ni–P/AMg6 coating was 14–16 µm thick and exhibited a pronounced nodular morphology with large agglomerates and a small number of isolated pores up to 300 nm in diameter. The 3–4 µm thick Ni–P/AM162H coating consisted of smaller globular features and agglomerates and contained both pore-free regions and localized clusters of pores approximately 1 µm in diameter. The Ni–P/Zircaloy-4 coating had a thickness of 4–6 µm and exhibited the most compact and uniform globular morphology, with no pores detected within the examined surface regions. XRD revealed broad Ni-based diffraction features for Ni–P/AMg6, consistent with a highly disordered or nanocrystalline structural state, whereas the coatings deposited on AM162H and Zircaloy-4 were identified as amorphous.

After normalization to a total Ni + P content of 100 wt.%, ICP-OES and EDS established the same compositional trend. The phosphorus contents determined by ICP-OES and EDS were 13 and 15 wt.% for Ni–P/AMg6, 4 and 5 wt.% for Ni–P/AM162H, and 9 and 9 wt.% for Ni–P/Zircaloy-4, respectively. The mechanical response did not exhibit a simple monotonic dependence on phosphorus content. Ni–P/AM162H showed the highest hardness and elastic modulus, 9.2±2.7 and 318±89 GPa, respectively, whereas Ni–P/Zircaloy-4 combined a hardness of 8.7±2.8 GPa with the highest indentation stiffness of 490 N/m. Ni–P/AMg6 exhibited the lowest hardness and elastic modulus, 4.1±1.4 and 186±52 GPa, respectively. The arithmetic mean roughness increased from 94 nm for Ni–P/AMg6 to 130 nm for Ni–P/AM162H and 420 nm for Ni–P/Zircaloy-4. Despite its greater roughness amplitude, the Zircaloy-4-based coating exhibited comparatively regular surface-profile variations, consistent with its densely packed globular morphology.

The electrochemical measurements showed that Ni–P/AM162H had the highest corrosion resistance under the applied test conditions, with a corrosion current of 2.0 µA and a corrosion rate of 0.3 mm/year. Ni–P/Zircaloy-4 exhibited closely comparable values of 2.0 µA and 0.3 mm/year, whereas Ni–P/AMg6 showed a substantially higher corrosion current of 170 µA and a corrosion rate of 4.2 mm/year. Because the polarization measurements lasted approximately 15 min, these values primarily characterize the initial corrosion behavior of the coatings. Scratch testing indicated satisfactory interfacial adhesion for Ni–P/AM162H and Ni–P/AMg6, although the latter exhibited more brittle cohesive cracking. In contrast, detachment of a comparatively large fragment from Ni–P/Zircaloy-4 indicated substantially weaker interfacial adhesion under the applied loading conditions.

Overall, AM162H and Zircaloy-4 are promising substrates for the formation of electroless Ni–P coatings intended for further development of UCN-source components, but each system requires specific optimization. Ni–P/AM162H combines high hardness and elastic modulus, the best corrosion resistance, and satisfactory adhesion; however, its low phosphorus content may limit its applicability in systems employing polarized UCNs. Ni–P/Zircaloy-4 combines a dense amorphous structure, high hardness, and corrosion resistance close to that of Ni–P/AM162H, but its interfacial adhesion requires improvement. Ni–P/AMg6 exhibits satisfactory adhesion and the highest phosphorus content but lower hardness, elastic modulus, and corrosion resistance. The results therefore provide a materials-science basis for further optimization of coating composition, surface preparation, deposition conditions, and interfacial adhesion. Assessment of long-term radiation stability and direct neutron-optical performance remains necessary before the coatings can be qualified for operation in UCN production and storage systems.

## Figures and Tables

**Table 1:** Specified chemical composition of the AMg6 aluminum–magnesium alloy according to GOST 4784-97 [52].

Element	Fe	Si	Mn	Ti	Al	Cu	Be	Mg	Zn	Other
Content (wt.%)	<0.40	<0.40	0.50–0.80	0.02–0.10	91.10–93.68	<0.10	0.0002–0.005	5.80–6.80	<0.20	<0.10

**Table 2:** Specified chemical composition of the AM162H aluminum–beryllium alloy according to the manufacturer’s specification [53].

Element or Constituent	Al	Be	O	C	Other Metallic Elements, Each
Content (wt.%)	Balance	60.0–64.0	≤1.0	≤0.1	≤0.2

**Table 3:** Chemical composition of the supplied Zircaloy-4 alloy according to the supplier’s documentation.

Element	Zr	Sn	Fe	Cr	O
Content (wt.%)	Balance	1.5	0.2	0.1	≤0.10

**Table 4:** Quantitative elemental composition of the Ni–P/AM162H coating.

Element Line	Weight, %	Weight Error, %
C	3.05	±0.10
P	4.31	±0.07
Ni	92.6	±0.5

**Table 5:** Quantitative elemental composition of the Ni–P/Zircaloy-4 coating.

Element Line	Weight, %	Weight Error, %
C	3.44	± 0.06
P	8.85	± 0.07
Ni	87.7	± 0.3

**Table 6:** Normalized Ni and P contents of the Ni–P coatings determined by ICP-OES and EDS.

Coating System	ICP-OES Ni (wt.%)	ICP-OES P (wt.%)	EDS Ni (wt.%)	EDS P (wt.%)
AMg6	87	13	85	15
AM162H	96	4	95	5
Zircaloy-4	91	9	91	9

Note: For each analytical method, the Ni and P contents were normalized to a total of 100 wt.%. Carbon detected by EDS was excluded from the normalization. Therefore, the reported values represent the relative Ni–P composition rather than the complete elemental composition of the coatings.

**Table 7:** Physicomechanical characteristics of the Ni–P coatings.

Coating System	Hardness, *H* (GPa)	Elastic Modulus, *E* (GPa)	Indentation Stiffness (N/m)
Ni–P/AMg6	4.1±1.4	186±52	480
Ni–P/AM162H	9.2±2.7	318±89	270
Ni–P/Zircaloy-4	8.7±2.8	212±58	490

**Table 8:** Surface roughness parameters of the Ni–P coatings.

Parameter	Unit	Ni–P/AMg6	Ni–P/AM162H	Ni–P/Zircaloy-4
Arithmetic mean roughness, $R_{a}$	nm	94	130	420
Root-mean-square roughness, $R_{\mathrm{ms}}$	nm	120	160	510
Ten-point profile height, $R_{z}$	nm	670	790	2400
Total profile-height range	nm	670	790	2400

**Table 9:** Corrosion parameters derived from the Tafel polarization curves.

Parameter	Ni–P/AMg6	Ni–P/AM162H	Ni–P/Zircaloy-4
Corrosion current, $I_{\mathrm{corr}}$ (μA)	170	2	2
Corrosion potential, $E_{\mathrm{corr}}$ (mV)	−740	−200	−240
Corrosion rate, $V_{\mathrm{corr}}$ (mm/year)	4.2	0.3	0.3

## Data Availability

The data presented in this study are available from the corresponding author upon reasonable request.

## Abbreviations

The following abbreviations are used in this manuscript:

UCN	Ultracold neutron(s)
VCN	Very cold neutron(s)
XRD	X-ray diffraction
SEM	Scanning electron microscopy
EDS	Energy-dispersive X-ray spectroscopy
INP	Institute of Nuclear Physics
JINR	Joint Institute for Nuclear Research
WWR-K	Water-Water Reactor–Kazakhstan
Ni-P	Nickel-phosphorus
